# Modelling intracellular competition for calcium: kinetic and thermodynamic control of different molecular modes of signal decoding

**DOI:** 10.1038/srep23730

**Published:** 2016-04-01

**Authors:** Gabriela Antunes, Antonio C. Roque, Fabio M. Simoes de Souza

**Affiliations:** 1 Laboratory of Neural Systems (SisNe), Department of Physics, Faculdade de Filosofia Ciências e Letras de Ribeirão Preto, Universidade de São Paulo, Ribeirão Preto, SP, Brasil; 2 Center for Mathematics, Computation and Cognition, Federal University of ABC, São Bernardo do Campo, SP, Brasil

## Abstract

Frequently, a common chemical entity triggers opposite cellular processes, which implies that the components of signalling networks must detect signals not only through their chemical natures, but also through their dynamic properties. To gain insights on the mechanisms of discrimination of the dynamic properties of cellular signals, we developed a computational stochastic model and investigated how three calcium ion (Ca^^2+^^)-dependent enzymes (adenylyl cyclase (AC), phosphodiesterase 1 (PDE1), and calcineurin (CaN)) differentially detect Ca^^2+^^ transients in a hippocampal dendritic spine. The balance among AC, PDE1 and CaN might determine the occurrence of opposite Ca^^2+^^-induced forms of synaptic plasticity, long-term potentiation (LTP) and long-term depression (LTD). CaN is essential for LTD. AC and PDE1 regulate, indirectly, protein kinase A, which counteracts CaN during LTP. Stimulations of AC, PDE1 and CaN with artificial and physiological Ca^^2+^^ signals demonstrated that AC and CaN have Ca^^2+^^ requirements modulated dynamically by different properties of the signals used to stimulate them, because their interactions with Ca^^2+^^ often occur under kinetic control. Contrarily, PDE1 responds to the immediate amplitude of different Ca^^2+^^ transients and usually with the same Ca^^2+^^ requirements observed under steady state. Therefore, AC, PDE1 and CaN decode different dynamic properties of Ca^^2+^^ signals.

With the expansion of our knowledge on cell signalling, it became evident that signal transduction emerges from the combination of highly interconnected networks often formed by signalling pathways with opposing actions, but activated by the same signal^1^. Consequently, the activity of different components of the signalling networks must be tailored to detect the chemical nature of the input signals and their dynamic features^2^,^3^. Among the processes that are likely to be regulated by dynamic characteristics of input signals are the long-term forms of synaptic plasticity.

Long-term forms of synaptic plasticity are persistent alterations in the efficacy of synaptic transmission, which can be depressed or potentiated^4^. The best characterized forms of long-term potentiation (LTP) and long-term depression (LTD) occur in the synapses between CA3 and CA1 hippocampal pyramidal neurons and require the activation of NMDA receptors (NMDARs), which promotes the influx of calcium ions (Ca^^2+^^) to the cytosol^1^,^4^. LTD induction involves low elevations of the Ca^^2+^^ concentration ([Ca^^2+^^]), which suggests the participation of molecules with high Ca^^2+^^ affinities^4^,^5^. LTP requires high [Ca^^2+^^] rises and, supposedly, requires the activation of molecules with moderate to weak Ca^^2+^^ affinities^4^,^5^. Therefore, it is generally accepted that the direction of the synaptic alteration, depression or potentiation, is determined by the relationship between the Ca^^2+^^ affinities of the molecules implicated with each type of plasticity and the magnitude of the [Ca^^2+^^] rise.

Among the molecules involved with LTP are several protein kinases, in contrast, LTD requires protein phosphatases^1^,^4^. Calcineurin (CaN) is the only phosphatase involved with plasticity activated by Ca^^2+^^ in its free form and complexed with calmodulin (Ca^^2+^^/CaM)^6^. During synaptic plasticity, CaN counteracts the activity of the cyclic adenosine monophosphate (cAMP)-dependent protein kinase (PKA)^7^. In the course of LTP, PKA phosphorylates the tyrosine phosphatase STEP and the residue Ser845 of AMPA receptors, both of which are dephosphorylated by CaN during LTD^7^.

In the hippocampus, PKA is regulated by Ca^^2+^^, but indirectly^8^,^9^. The activation of PKA involves its binding to cAMP^10^ produced by adenylyl cyclases (ACs). Two types of AC, 1 and 8, are stimulated by Ca^^2+^^/CaM^11^. AC1, referred as AC in the remaining of the paper, is neuron-specific and concentrated at the axons and dendrites, but also expressed at the synapses^12^. To counteract the activity of AC, a specific phosphodiesterase, PDE1A2 (referred as PDE1 in the remaining of the paper), the enzyme that hydrolyses cAMP, is highly concentrated in the brain and is also regulated by binding to Ca^^2+^^/CaM^8^.

The rise of Ca^^2+^^ and the subsequent elevation of cAMP activates PKA during LTP, but also activates Epac, a molecule involved with LTD^13^. Thus, [Ca^^2+^^] elevations activate, among other targets, CaN, a phosphatase essential for LTD, AC and PDE1, both of which regulate the level of cAMP, a second messenger implicated with LTD and LTP. In such a scenario, it is very difficult to predict how the Ca^^2+^^ signals are decoded to ensure the occurrence of specific forms of synaptic plasticity. AC, CaN, and PDE1 have Ca^^2+^^ requirements below 1 μmol.L^−1 ^6,8,14, and can potentially be activated by protocols of LTP and LTD induction. Thus, in this work, we investigated whether the activations of AC, PDE1 and CaN, are regulated by other properties besides the amplitude of the Ca^^2+^^ signals. We developed a stochastic computational model of the detailed mechanisms of activation of each one of these enzymes placed inside a hippocampal dendritic spine containing comprehensive mechanisms of Ca^^2+^^ dynamics. This model was used to investigate how CaN, AC and PDE1 decode simple and complex Ca^^2+^^ transients to promote specificity among their activations. Our results demonstrated that these enzymes have different modes of Ca^^2+^^ signals decoding that arise from thermodynamic and kinetic factors.

## Results

### Validation of the model

**Figure 1:**
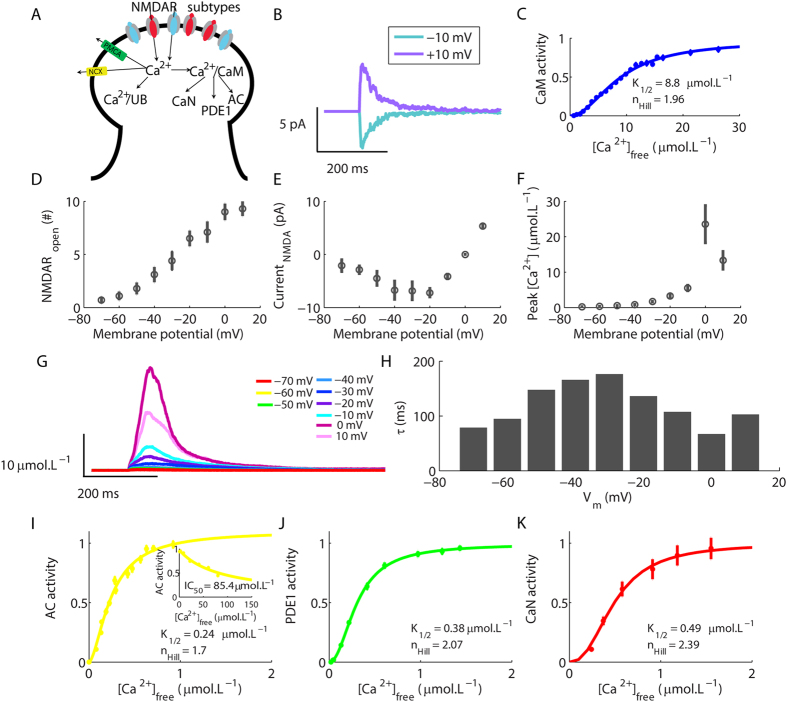
Validation of the components of the model. (**A**) Scheme of the dendritic spine with the model components. The Ca^^2+^^ influx is mediated by NMDARs and the efflux is generated by NCX and PMCA. Intracellular Ca^^2+^^ binds to unspecific buffers (Ca^^2+^^/UB) and to CaM (Ca^^2+^^/CaM) that activates the enzymes AC, PDE1, and CaN. (**B**) Time courses of the NMDAR-mediated current evoked by a brief pulse of glutamate at different V_m_. Each curve shows the mean result of 10 single runs of the model. (**C**) Dose-response curve for the formation of the complex (Ca^^2+^^)_4_CaM. Each dot shows the mean (±sem) result of 10 simulations. (**D**) Maximum number of open NMDAR channels evoked by single pulses of glutamate as a function of V_m_. (**E**) Peak NMDAR-mediated currents evoked by single glutamate pulses as a function of V_m_. (**F**) Maximum rises of [Ca^^2+^^] evoked by single glutamate pulses at different V_m_. Each dot in Fig. D–F shows the mean result of 10 simulations. (**G**) Mean time courses of the synaptically evoked Ca^^2+^^ transients as a function of V_m_ calculated from 10 runs of the model. (**H**) Values of τ for the decay of the Ca^^2+^^ transients showed in (**G**). (**I**–**K**) Sigmoidal dose-response relationships of AC (**I**), PDE1 (**J**) and CaN (**K**) activations as a function of [Ca^^2+^^] in presence of saturating CaM. Each dot shows the mean result of 10 simulations.

We developed a computational model of a single hippocampal spine, the cellular compartment where the glutamatergic postsynaptic machinery is located^15^. Because of the small volume of the spines (~fL), the model was solved stochastically^16^. In our simulated system, stochasticity was fundamental to capture realistic patterns of Ca^^2+^^ transients, which are highly variable^17^. The model consisted of the three enzymes investigated (AC, PDE1 and CaN), and detailed mechanisms of Ca^^2+^^ dynamics formed by its influx through NMDARs channels, buffering and extrusion (Fig. 1A).

Structurally, NMDARs are assemblies of two GluN1 and two GluN2 subunits^18^. In the hippocampus, GluN2A and GluN2B are the predominant GluN2 subunits^19^,^20^,^21^. We implemented the NMDAR population composed of two types of receptors with a specific ratio (65% GluN1/GluN2A and 35% GluN1/GluN2B receptors)^18^,^19^,^20^. We simulated the NMDARs with the same kinetic model, but different sets of rate constants^21^. Figure 1B shows some examples of the time courses of the NMDARs current evoked by a single glutamate pulse (1 mmol.L^−1^ of magnitude and 1 ms of duration).

To reproduce physiological changes of [Ca^^2+^^], two buffers were simulated, an unspecific buffer (UB) and CaM. We simulated CaM according to its structure composed by two distinct domains, each one containing a pair of Ca^^2+^^-binding sites^22^. We validated the model of association of Ca^^2+^^ to CaM by fitting a dose-response curve with the Hill equation:


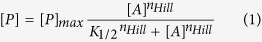


where [P] is the activated protein, [P]_*max*_ is its maximum activation, [A] is the concentration of its activator, n_Hill_ is the Hill coefficient, and K_1/2_ is the concentration of activator required to activate the half maximum concentration of [P]. The n_Hill_ and K_1/2_ obtained by fitting the dose-response curve of activation of CaM as a function of free [Ca^^2+^^] ([Ca^^2+^^]_free_) (Fig. 1C) were in accordance with published values^22^.

We verified the accuracy of the Ca^^2+^^ dynamics implemented in the computational model through its ability to reproduce synaptically evoked Ca^^2+^^ transients observed experimentally. NMDARs have a very characteristic current (I)-membrane potential (V_m_) relationship caused by the voltage-dependent blockage of their channels by magnesium ions (Mg^^2+^^)^23^. To validate the synaptically evoked Ca^^2+^^ signals, first we verified the number of open NMDAR channels and the NMDAR-mediated current evoked by a glutamate pulse (amplitude of ~700 μmol.L^−1^ and duration of 1 ms) as a function of V_m_. The number of open NMDAR varied with V_m_ (Fig. 1D), and the current-V_m_ relationship obtained with the model exhibited the typical negative slope from −70 to approximately −35 mV caused by the Mg^^2+^^ blockage (Fig. 1E)^23^,^24^. We simulated the influx of Ca^^2+^^ through the open Mg^^2+^^-unblocked NMDARs considering that the fractional Ca^^2+^^ current changes as a function of V_m _25 (see Supplementary Information for details). The peak amplitudes of the Ca^^2+^^ rises were approximately 0.2 μmol.L^−1^ and 5 μmol.L^−1^ at −70 mV and −10 mV, respectively, but the maximum [Ca^^2+^^] elevations were obtained at 0 mV, which corresponds to the V_m_ in which the NMDARs exhibit their maximum fractional Ca^^2+^^ current (Fig. 1F,G)^24^,^25^. Typical values of Ca^^2+^^ rises described in the literature range from nmol.L^−1^ to several μmol.L^−1^ depending of V_m _26, and these magnitudes can vary up to ten-fold in the spines^17^. The time constants for the Ca^^2+^^ decay (τ, calculated as a monoexponential) obtained for our simulations varied from 77 to 177 ms. Experimental works have reported values ranging from 89 ms^26^ to approximately 200 ms^27^.

As mentioned previously, CaM has four Ca^^2+^^-binding sites that, when filled, promote a conformational change that exposes sites for target interactions^28^. This conformational change can occur when CaM is associated to less than four Ca^^2+^^, but the binding of all four ions stabilizes its open conformation^28^. However, CaM partially loaded with Ca^^2+^^ can interact with AC, PDE1, and CaN^11^,^29^,^30^, which was implemented in our model. For clarification, we used two terms to refer to complexes formed between Ca^^2+^^ and CaM: (Ca^^2+^^)_4_CaM denotes CaM loaded with four Ca^^2+^^, and Ca^^2+^^/CaM refers to all complexes of CaM with four or less Ca^^2+^^.

To validate the reactions and parameters used to simulate PDE, AC, and CaN, we simulated each one of them isolated from the other components of the model and, in the presence of saturating concentrations of CaM, we varied [Ca^^2+^^] systematically. We performed the simulations until the system had reached equilibrium. After that, we annotated the remaining [Ca^^2+^^]_free_ and the concentration of the activated molecule under analysis to plot its dose-response curve of activation. To validate AC, we verified its activation as a function of [Ca^^2+^^] in the presence of saturating CaM (Fig. 1I, 1 μmol.L^−1^ of AC to 10 μmol.L^−1^ of CaM). The parameters obtained by fitting the equation (1) were consistent with experimental data (K_1/2_ of ~0.2 μmol.L^−1^ and n_Hill_ of 2)^14^. Ca^^2+^^ not only stimulated AC, but also inhibited its activity when present in high concentrations (Fig. 1I inset), as observed experimentally (IC_50_ ~80 μmol.L^−1^)^31^.

We validated the model of PDE1 through its dose-response curve of activation as a function of [Ca^^2+^^] with saturating CaM (1 μmol.L^−1^ of PDE1 to 10 μmol.L^−1^ of CaM, Supplementary Fig. S1A shows the results for other concentrations of CaM). The results obtained by fitting equation (1) were in accordance with experimental data (K_1/2_ of approximately 0.35 μmol.L^−1^ and n_Hill_ around 2)^8^,^32^ (Fig. 1J).

CaN is a heterodimer composed by a catalytic subunit (CNA) with a Ca^^2+^^/CaM-binding site, and a regulatory subunit (CNB) that contains four Ca^^2+^^-binding sites^6^,^33^. The association of Ca^^2+^^ to CNB is a prerequisite for the binding of Ca^^2+^^/CaM to CNA^6^,^34^,^35^. We validated the model of CaN through its global Ca^^2+^^ requirement in presence of saturating CaM (1 μmol.L^−1^ of CaN to 10 μmol.L^−1^ of CaM, Supplementary Fig. S1B shows the dose-response curve of CaN in absence of CaM). The parameters obtained by fitting equation (1) were consistent with experimental observations (K_1/2_ of 0.5–0.8 μmol.L^−1^ and n_Hill_ around 2.5–3) (Fig. 1K)^6^,^36^.

Elevations of Ca^^2+^^ lead to the formation of Ca^^2+^^/CaM, which activates AC, PDE and CaN. The inactivation rate constants of each protein after a 1 s pulse of Ca^^2+^^ of 50 μM were 33.92 s^−1^ for Ca^^2+^^/CaM, 4.97 s^−1^ for PDE, 1.09 and 6.05 s^−1^ for AC, and 3.67 s^−1^ for CaN. We fitted the inactivation rate constants of AC with a biexponential function, and used a monoexponential function for the other species. The values obtained are in close agreement with experimental data. For instance, the rate of inactivation of CaN was estimated in 4 s^−1^ with a slow component of 0.4 s^−1 ^37. The rates of Ca^^2+^^ dissociation from CaM in the presence of AC indicated that AC inactivation has two slow components estimated in 8 s^−1^ and 1 s^−1 ^11. The inactivation of CaM has a fast and a slow component. At 3° to 22 °C, the slow component of CaM inactivation has been estimated around 2 s^−1^ and 10 s^−1^, respectively^38^, and is faster at more physiological temperatures^39^.

The dose-response curves obtained with the models of AC, PDE1 and CaN demonstrated that, while CaM requires a high elevation of [Ca^^2+^^] to become fully saturated (~20 μmol.L^−1^), the maximum activations of its targets involve lower [Ca^^2+^^] rises (~0.5–1 μmol.L^−1^)^6^,^8^,^14^. Thus, the presence of AC, PDE1 and CaN increases the affinity of CaM for Ca^^2+^ ^11,30,40, as implemented in our computational model, which determines that the Ca^^2+^^ dependency of CaM in its native environment reflects the relative concentrations of all of its potential competing targets^41^.

A common aspect of AC, PDE1 and CaN activations is their low Ca^^2+^^ requirements under steady state indicated by their K_1/2_s (Fig. 1I–K). Consequently, if the amplitudes of the Ca^^2+^^ signals were the crucial factor to determine the induction of LTD or LTP, which involve, respectively, low (~750 nmol.L^−1^) and high (>10 μmol.L^−1^) rises of [Ca^^2+^^]^42^, a large amount of AC, PDE1 and CaN would be activated during both forms of synaptic plasticity. However, differentially from the steady state conditions typically used to determine dose-response relationships, the Ca^^2+^^ signals in the intracellular environment are continuously changing over time^26^.

### Temporal decoding of Ca^^2+^^ signals

The activation and inactivation of proteins can occur under thermodynamic or kinetic control, which can lead to striking different outcomes. When a system is under thermodynamic control (steady state), the concentrations of the competing species are determined by the stability difference among them. The most stable species are present in larger amounts. If a system is not in steady state, the formation of competing species is governed by competing rates (kinetic control), and the species formed faster are present in larger concentrations.

Most biological systems are incessantly changing over time and operate far from steady state. Thus, it is unlikely that the activations of AC, PDE1 and CaN in their native environment are determined exclusively by thermodynamic factors. Consequently, it is impossible to predict their levels of activation for a given [Ca^^2+^^] rise considering exclusively their Ca^^2+^^ requirements obtained under steady state. Based on this aspect, the next step in our work focused on the analysis of the activations of AC, PDE1, and CaN when stimulated by Ca^^2+^^ transients.

To verify how Ca^^2+^^ transients regulate the levels of activation of AC, PDE1 and CaN, they were simulated in the hippocampal spine with equimolar concentration (1 μmol.L^−1^), in the presence of saturating CaM (40 μmol.L^−1^) and were stimulated by trains of five Ca^^2+^^ pulses with different amplitudes (ranging from 0.08 μmol.L^−1^ to approximately 50 μmol.L^−1^), durations (1–30 s) and time intervals (1 s (1 Hz) and 100 ms (10 Hz)). To each Ca^^2+^^ pulse within a train, we verified the peak activation of the molecules analysed obtained as a function of the peak [Ca^^2+^^]. As the three enzymes are activated by Ca^^2+^^ by binding to Ca^^2+^^/CaM, we also included the formation of (Ca^^2+^^)_4_CaM in our analysis. We used the peak activations of the molecules analysed obtained for each Ca^^2+^^ signal to plot curves of activation *versus* the peak amplitude of [Ca^^2+^^]. For that, we annotated the maximum activation of the molecules analysed during the occurrence of a Ca^^2+^^ pulse and the corresponding peak [Ca^^2+^^]. The dose-responses curves of maximum activations as functions of the peak [Ca^^2+^^] were fitted using equation (1). A prior work analysed the activation of enzymes integrated over time (area of the curves of activation), which is important to take into account differences in the time courses of activations^43^. However, we have opted to analyse exclusively the peak concentrations to compare the apparent Ca^^2+^^ requirements obtained for dynamic patterns of Ca^^2+^^ stimulation with the values observed under steady state.

**Figure 2:**
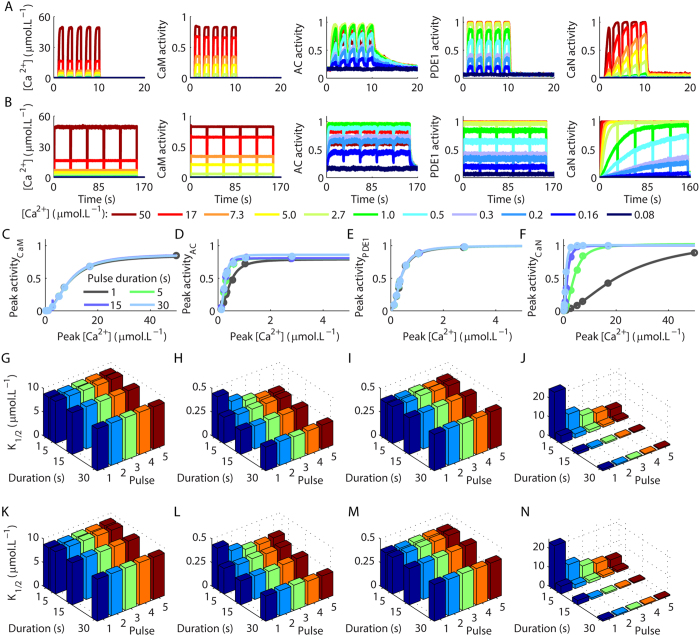
Activation of CaM, AC, PDE1 and CaN as a function of the peak amplitudes of Ca^^2+^^ transients. (**A**,**B**) Average time courses of CaM, AC, PDE1 and CaN activations evoked by trains of Ca^^2+^^ pulses with 1 s (**A**) and 30 s (**B**) of duration released with 1 s of inter pulse interval. (**C**–**F**) Sigmoidal dose-response curves of the peak activations of CaM (**C**), AC (**D**), PDE1 (**E**) and CaN (**F**) activity as a function of the peak concentration of the first Ca^^2+^^ pulse of a train with five pulses (the dose-responses curves for the other pulses are showed in Supporting Information Fig. S4). (**G**–**N**) K_1/2_ calculated from the curves showed in (**C**–**F**) and in the Supporting Information Figs S3–S5, for the activations of CaM (**G**,**K**), AC (**H**,**L**), PDE (**I**,**M**), CaN (**J**,**N**) evoked by distinct number of Ca^^2+^^ pulses released with different inter pulse interval (**G**–**J**): 100 ms, (**K**–**N**): 1 s), and with durations varying from 1 s to 30 s.

The curves obtained for the formation of (Ca^^2+^^)_4_CaM demonstrated that the activity of CaM was independent of the durations of the Ca^^2+^^ pulses tested and of the number of pulses used to stimulate the model (Fig. 2A–C,G,K, Supplementary Figs S2–S5). All the curves exhibited similar values of K_1/2_ (Fig. 2G,K) and n_Hill_ (Supplementary Fig. S5A,E) independently of the durations of the Ca^^2+^^ transients, the number of Ca^^2+^^ pulses and the inter pulse interval, and these parameters were very similar to the values obtained under steady state (Fig. 1C).

AC presented a nonlinear change in its Ca^^2+^^ requirement as a function of the durations of the Ca^^2+^^ pulses (Fig. 2A,B,D, Supplementary Figs S2–S5). The values of K_1/2_ of AC decreased as the durations of the Ca^^2+^^ pulses were increased (Fig. 2H,L), but converged to the result obtained under steady state (Fig. 1I) when the model was stimulated with Ca^^2+^^ signals longer than 5 s. The number of Ca^^2+^^ pulses and the inter pulse interval of the signals used to activate it were important to reduce its apparent Ca^^2+^^ requirement evoked by brief signals (1 s), but had no effect on its activation when longer transients were used as input signals (Fig. 2A,B,D, Supplementary Figs S2–S5).

PDE activation showed no variation in its Ca^^2+^^ requirement for the durations of Ca^^2+^^ pulses tested, the number of pulses or the inter pulse interval of the input signals (Fig. 2A,B,E, Supplementary Figs S3–S5). Both the values of K_1/2_ (Fig. 2I,M) and n_Hill_ (Supplementary Fig. S5C,G) were similar to the results obtained under steady state (Fig. 1J).

CaN had its [Ca^^2+^^] requirement regulated by the duration and the number of Ca^^2+^^ pulses used as input-signals of the model (Fig. 2A,B,F, Supporting Information Figs S3–S5). The K_1/2_s of CaN decreased with the increase in the durations of the Ca^^2+^^ pulses (Fig. 2J). The K_1/2_s converged to the result obtained under steady state only when the model was stimulated with at least two Ca^^2+^^ pulses of 30 s or four pulses of 15 s of duration. The frequencies of the Ca^^2+^^ pulses tested had little effect on CaN activity. The values of n_Hill_ obtained with the sigmoid curves indicate that the durations of the Ca^^2+^^ pulses slightly affected this parameter (Supplementary Fig. S5D,H).

The ability of CaN and AC to exhibit different Ca^^2+^^ requirements as a function of the durations and number of Ca^^2+^^ signals, at least within a range, indicated that they can temporally decode brief Ca^^2+^^ transients because, under these circumstance, their interactions with Ca^^2+^^ occur under kinetic control. Consequently, they can present different levels of activation according to the duration of the Ca^^2+^^ signals or the number of pulses used as inputs of the model. The next stage of our work consisted in simulating the activation of these molecules when stimulated by complex physiological Ca^^2+^^ signals.

### Decoding synaptically evoked Ca^^2+^^ transients

LTP and LTD are typically induced by glutamate pulses released at high and low frequencies, respectively^4^. To verify whether and how AC, PDE1 and CaN decode the physiological Ca^^2+^^ signals evoked by trains of synaptic stimulation, glutamate pulses released at different frequencies (100 pulses at 10 and 1 Hz) were used to stimulate the model of the hippocampal spine and promote the Ca^^2+^^ influx through NMDAR channels. As the opening of NMDARs requires both the binding of glutamate and the depolarization of the postsynaptic cellular membrane^18^, for each frequency tested we simulated different values of clamped V_m_ to produce physiological Ca^^2+^^ signals with different amplitudes.

**Figure 3:**
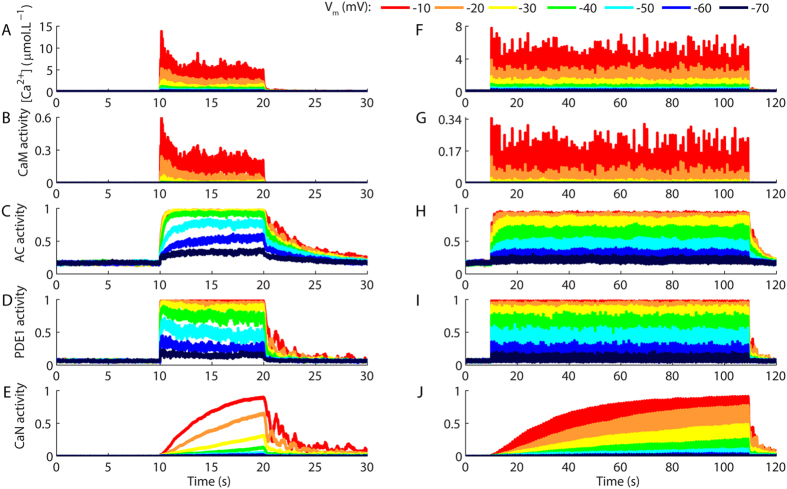
Synaptically evoked activations of different components of the model. (**A**–**E**) Ca^^2+^^ signals (**A**) and the resulting activations of CaM (**B**), AC (**C**), PDE1 (**D**), and CaN (**E**) evoked by 100 pulses of glutamate released at 10 Hz with clamped V_m_ ranging from −10 mV to −70 mV. (**F**–**J**) Ca^^2+^^ transients (**F**) and activations of CaM (**G**), AC (**H**), PDE1 (**I**), and CaN (**J**) evoked by 100 pulses of glutamate released at 1 Hz. Each curve corresponds to mean results of 10 runs of the model. The glutamate trains used to stimulate the model are showed in Supplementary Fig. S6A–D.

Stimulations of the model with 100 glutamate pulses at 10 Hz produced complex Ca^^2+^^ signals (Fig. 3A), which reflected the desensitization of NMDARs (Supplementary Fig. S6). CaM activity mimicked the shape of the Ca^^2+^^ signals (Fig. 3B) and induced fast and high levels of AC (Fig. 3C) and PDE1 activations (Fig. 3D), which reached steady levels of activity with very few pulses of stimulation. In contrast, CaN activity exhibited a pronounced summation and was clearly regulated by the number of Ca^^2+^^ pulses as recently demonstrated experimentally^44^. Comparatively, stimulations at 100 Hz promoted lower activations of CaN for Ca^^2+^^ pulses with much higher peak amplitudes (Supplementary Fig. S7). In addition, protocols of spike-timing dependent plasticity (STDP) consisting of stimulation released at 5 Hz (sixty pulses of glutamate paired with spike trains) promoted low activations of CaN, but strong activations of AC and PDE1 (Supplementary Fig. S8).

Stimulations of the model with 100 glutamate pulses at 1 Hz resulted in rises of Ca^^2+^^ (Fig. 3F) and the activations of CaM (Fig. 3G) without the decay caused by the desensitization of NMDARs. However, AC activations were smaller (Fig. 3H) for V_m_s between −70 to −30 mV in comparison to the results for 10 Hz, which indicated that the large inter pulse intervals of the Ca^^2+^^ signals reduced the summation of its activity. PDE1 activations were high for all V_m_ values tested (Fig. 3I). In contrast to AC, glutamate pulses at 1 Hz promoted stronger activations of CaN in comparison to the pulses released at 10 Hz and 100 Hz (Fig. 3J, Supplementary Fig. S7).

**Figure 4:**
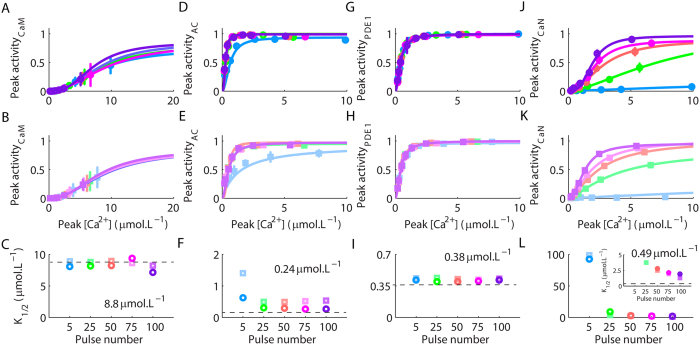
Dynamic changes in the Ca^^2+^^ required for the activations of the components of the model during trains of stimulation. Sigmoid curves and the respective values of K_1/2_ estimated for the maximum activities of CaM (**A**–**C**), AC (**D**–**F**), PDE1 (**G**–**I**), and CaN (**J**–**L**), as functions of the peak rises of Ca^^2+^^ produced by the fifth, the twenty fifth, the fiftieth, the seventieth fifth, and the one hundredth pulse of glutamate released at 10 Hz (squares) and 1 Hz (circles). Dashed lines indicate the original K_1/2_ obtained under steady state, their exact values are written in the panels. The dashed line in panel L is indicated in the inset that expanded the scale for better visualization. For each frequency tested, different values of clamped V_m_ were used to produce Ca^^2+^^ signals with different peak amplitudes (Fig. 3).

Next, we verified whether the Ca^^2+^^ requirements of CaM, AC, PDE1, and CaN were dynamically changing during the trains of stimulation shown in Fig. 3. We analysed their peak activations as functions of the maximum amplitudes of [Ca^^2+^^] caused by the fifth, the twentieth-fifth, the fiftieth, the seventieth-fifth, and the one-hundredth glutamate pulse released at 10 and 1 Hz. Thus, for each Ca^^2+^^ pulse analysed, we annotated the corresponding peak activation of CaM, AC, PDE1 and CaN and verified the dynamic fluctuations in the Ca^^2+^^ requirements during their stimulations. The results obtained were used to plot dose-response curves fitted with equation (1). Figure 4A,B show the peak activations of CaM as a function of Ca^^2+^^ transients caused by the glutamate pulse numbers 5, 25, 50, 75 and 100 released at 10 Hz (Fig. 4A) and 1 Hz (Fig. 4B) for different V_m_s. These results demonstrated that the Ca^^2+^^ required to form (Ca^^2+^^)_4_CaM (indicated as CaM activity in the panels) remained unchanged during the trains of stimulation. Consequently, all the curves were superposed, and their corresponding K_1/2_s (Fig. 4C) were similar to the result obtained under steady state (Fig. 1C), which suggested that, during the trains of stimulation, the interaction of CaM with Ca^^2+^^ occurred under thermodynamic control.

AC exhibited a dynamic change in its Ca^^2+^^ requirement as a function of the numbers of successive Ca^^2+^^ signals used to stimulate it (Fig. 4D–F). The Ca^^2+^^ requirements of AC, measured through the K_1/2_s, approached the value obtained under steady state after 25 glutamate pulses at 10 Hz. However, for glutamate pulses at 1 Hz, the K_1/2_s of AC were always higher (the smallest value was 0.53 μmol.L^−1^) than the K_1/2_ observed under steady state (Fig. 1I). Therefore, AC discriminated the number of Ca^^2+^^ pulses used to stimulate its activation and dynamically changed its Ca^^2+^^ requirements during the trains of stimulation, but it was also sensitive to the inter pulses intervals of the Ca^^2+^^ transients.

Our results demonstrated a slight increase in the K_1/2_s of PDE1 activation for glutamate stimulations at 1 Hz, but, for stimulations at 10 Hz, the K_1/2_s were consistent with the value observed under steady state (Figs 1J and 4G–I).

CaN presented a dynamic change in its Ca^^2+^^ requirement as a function of different numbers of successive Ca^^2+^^ pulses (Fig. 4J–L). However, the Ca^^2+^^ requirement of CaN converged to similar values during the trains of stimulation independently of the frequency used. Nevertheless, for both frequencies tested, the values of K_1/2_ were always higher (Fig. 4L inset) in comparison to the value estimated under steady state (Fig. 1K), indicating that the interactions between Ca^^2+^^ and CaN in these situations always occurred under kinetic control. Therefore, the Ca^^2+^^ dependency of CaN was modulated by a combination of peak amplitude, duration and number of Ca^^2+^^ pulses, which suggested that CaN integrated the amount of available Ca^^2+^^ over time.

**Figure 5:**
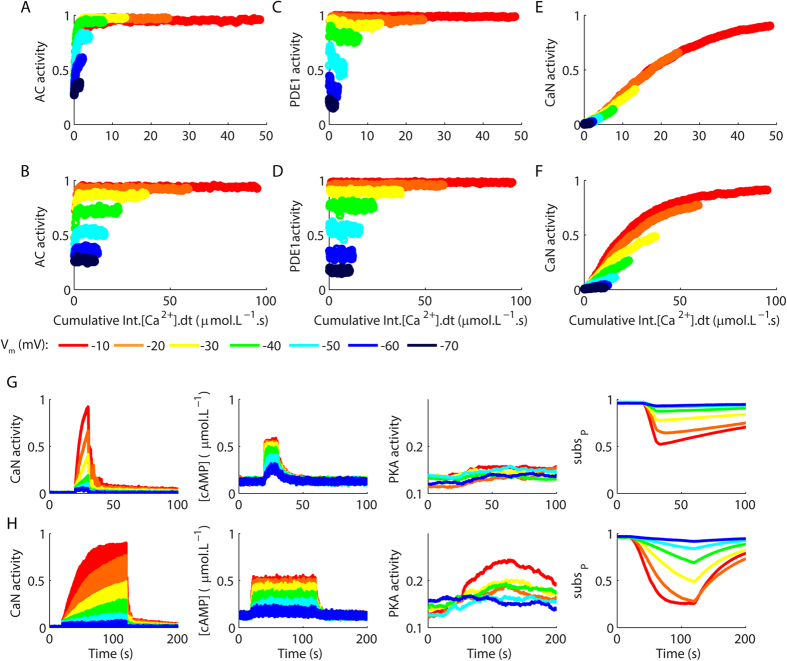
Activations of the components of the model as functions of the cumulative integration of the Ca^^2+^^ signals over time and the consequences of the different mechanisms of Ca^^2+^^ dynamics to other molecules. (**A**,**B**) Activations of AC obtained as functions of the cumulative temporal integrations of Ca^^2+^^ transients evoked by 100 glutamate pulses released at 10 Hz (**A**) and 1 Hz (**B**). (**C**,**D**) Activations of PDE1 observed for the cumulative temporal integrations of Ca^^2+^^ signals produced by 100 glutamate pulses released at 10 Hz (**C**) and 1 Hz (**D**). (**E**,**F**) Relationships between the activations of CaN and the cumulative temporal integrations of the Ca^^2+^^ signals produced by 100 glutamate pulses released at 10 Hz (**E**) and 1 Hz (**F**). (**G**,**H**) Dephosphorylation of a hypothetical target (subs^P^) consequent to the competing action of CaN and PKA, which is regulated by the level of cAMP controlled by AC and PDE1. The stimulations used in this simulations consisted of 100 pulses of glutamate released at 10 Hz (**G**) and 1 Hz (**H**) as in Fig. 3. The curves are averages of 10 simulations.

To verify whether CaN, AC and PDE1 integrate the simulated Ca^^2+^^ signals, we plotted their activations, obtained from Fig. 3, as functions of the cumulative Ca^^2+^^ signals integrated over time for the frequencies of glutamate tested. AC activations escalated with the cumulative Ca^^2+^^ signals integrated over time for glutamatergic stimulations at 10 Hz, but this relationship was less pronounced for stimulations at 1 Hz (Fig. 5A,B). These results indicated that AC discriminated the frequencies of Ca^^2+^^ stimulation by integrating signals with efficiencies that depended of the inter pulse interval. In contrast, PDE1 activations were not regulated by the cumulative Ca^^2+^^ signals integrated over time (Fig. 5C,D). CaN integrated the Ca^^2+^^ signals produced by all the frequencies of glutamate trains tested (Fig. 5E,F).

What are the consequences of these different modes of signal integration? In signalling pathways, the combined patterns of activations of CaN, AC and PDE1 are likely to propagate to other molecules and entail the occurrence of several different outcomes. To verify some of the putative consequences of the mechanism of signal integration observed in this work to other signalling molecules, we expanded our model to include the production and degradation of cAMP by AC and PDE1, respectively, and the consecutive activation of PKA, a kinase that typically counteract CaN activity. The reactions and parameters used to simulate the catalytic activity of AC and PDE and the activation of PKA are described in the Supplementary Information. We validated the model of PKA thermodynamically and kinetically based on comparisons with experimental data (Supplementary Fig. S9). Then, we implemented a generic substrate (subs) in the model that was phosphorylated by PKA (subs^P^) and dephosphorylated by CaN. We used the same rate constants for the reactions of phosphorylation and dephosphorylation to facilitate the comparisons between PKA and CaN. Under basal conditions, PKA has a higher activity than CaN^45^. Because of that, the simulations began with the total amount of subs completely phosphorylated and we verified its dephosphorylation caused by the trains of 100 pulses of glutamate at 10 Hz and 1 Hz described previously. Our results demonstrated that the concentration of cAMP ([cAMP]) changed symmetrically with [Ca^^2+^^], but promotes low levels of PKA activation (Fig. 5G,H). We attributed the low activation of PKA to the strong regulation that the concentration of its substrates exert on it^46^ (we used 100 μmol.L^−1^ of phosphorylated subs to 1 μmol.L^−1^ of PKA, during experiments *in vitro*, strong activations of PKA are observed with 1000-fold excess of non-phosphorylated substrate^46^), and, in addition, to the rises of [cAMP] observed, which can be much higher when AC is stimulated with Ca^^2+^^ in combination with protein G_αs _47. Nevertheless, we verified that the model exhibited very different levels of phosphorylated substrate (subs^P^) for the protocols tested. Thus, we observed stronger levels of dephosphorylation of subs^P^ for stimulations at 1 Hz in comparison to 10 Hz (Fig. 5G,H). Therefore, our results indicated that the different patterns of activations of AC, PDE1 and CaN regulated by the dynamic properties of the input signals can propagate in a nonlinear manner to other components of the signalling pathways.

### Molecular mechanisms for different modes of signal decoding

**Figure 6:**
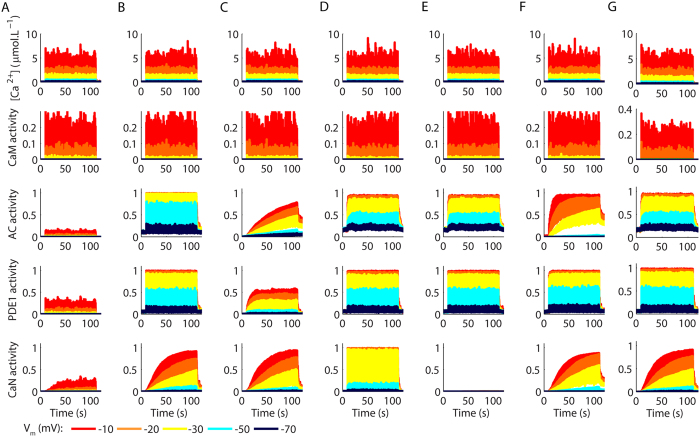
Molecular mechanisms that regulate the decoding of Ca^^2+^^ signals in the model. (**A**) Results of a modified version of model that lacks the increase in the affinity of Ca^^2+^^ for CaM caused by the presence of AC, PDE1, and CaN. (**B**,**C**) Effects of a 100-fold increase (**B**) and a 100-fold decrease (**C**) in the rate constants for the binding and unbinding of CaM to AC, PDE1, and CaN. (**D**,**E**) Consequences of a 100-fold increase (**D**) and a 100-fold reduction (**E**) in the rate constants for the binding and unbinding of Ca^^2+^^ to the subunit CNB of CaN. (**F**) Effects of the omission of the interactions of AC, PDE, and CaN with CaM partially loaded with Ca^^2+^^ in the model. (**G**) The results of the original model were replotted from Fig. 3F–J for comparisons. All panels shown the results of the model for stimulations composed by 100 pulses of glutamate with 1 ms of duration and released at 1 Hz. During the simulations, we clamped V_m_ at different values to promote distinct Ca^^2+^^ rises.

What are the mechanisms responsible for the different types of signal integration observed? It is widely known that an important property of the interaction of CaM with its targets is that they usually alter its affinity for Ca^^2+^ ^40,41, as simulated in our work. To verify whether the alterations in the affinity of CaM for Ca^^2+^^ caused by CaM targets contributed to the patterns of activations observed in Fig. 3, we removed them from the model and performed simulations using trains of glutamate at 1 Hz as in Fig. 3F–J (these results are replotted in Fig. 6G for comparison). The results obtained demonstrated that the changes in the affinities for the binding of CaM to Ca^^2+^^ caused by CaM targets control their magnitudes of activation (Fig. 6A).

Next, we investigated the contributions of kinetic factors for the time courses of activity showed in Fig. 3. We simultaneously altered the rate constants for the binding and unbinding of CaM with its targets. These alterations consisted in a 100-fold increase (Fig. 6B) and a 100-fold reduction in their original values (Fig. 6C). The increase in the velocity for the binding and unbinding of CaM altered slightly the patterns of activation of AC for some of the V_m_ tested, but had no effect on the activities of PDE1 and CaN (Fig. 6B). In contrast, the reduction in the velocity for the interactions of CaM to AC and PDE1 had a remarkable effect on their time courses of activations, but a minor effect on the activity of CaN (Fig. 6C).

CaN interacts directly with Ca^^2+^^ prior to its binding to CaM^6^,^34^,^35^. In consequence, these Ca^^2+^^ binding events are putative bottlenecks for CaN activation. To test this possibility, we performed simulations of the model with altered rate constants for the association/dissociation of Ca^^2+^^ to CNB. A 100-fold increase (Fig. 6D) or a 100-fold reduction (Fig. 6E) in the rate constants for the binding and unbinding of Ca^^2+^^ to CNB had profound effects on its time courses and magnitudes of activation (Fig. 6D,E). In fact, the reduction in the velocity for the interaction of CaN to Ca^^2+^^ abolished its activation almost completely (Fig. 6E). Experimental data indicated the interaction of Ca^^2+^^ to CaN is slow^48^, which imposes a significant constrain to CaN activation evoked by brief signals. Thus, in our model, it was the Ca^^2+^^ requirement of CaN instead of its interaction with Ca^^2+^^/CaM that determined its patterns of activation.

The last aspect of our model that we investigated was the interactions of AC, PDE1 and CaN with CaM partially loaded with Ca^^2+^^, which is supposed to contribute to their activations. To verify how much the interactions of AC, PDE1 and CaN with CaM partially loaded with Ca^^2+^^ regulate their activations, we removed these interactions from the model. Then, we performed simulations of the model stimulated with glutamate pulses (100 pulses of 1 ms of duration released at 1 Hz). The results demonstrated the binding of CaM partially loaded with Ca^^2+^^ to AC was important to determine its pattern of activation, but had smaller effects on the activations of CaN and PDE1 (Fig. 6F). PDE1 interacts with CaM preferentially associated to Ca^^2+^^ through the Ca^^2+^^ binding sites located in its N-terminal, which have weaker Ca^^2+^^ affinities in comparison to the sites of its C-terminal^38^. Consequently, states of CaM loaded with Ca^^2+^^ only in its N-terminal were rare in our model and contributed little to PDE1 activation. The results observed for CaN confirmed that the bottleneck for its activation in our model was mostly its interaction with Ca^^2+^^. Changes in the parameters and mechanisms of the interaction between CaM and CaN had only minor consequences to its pattern of activation.

## Discussion

In this work, we developed a stochastic computational model of CaN, AC and PDE1 to verify how they decode different Ca^^2+^^ signals. These enzymes were simulated with a great level of details and considering how their activations with Ca^^2+^^/CaM affect the CaM affinity for Ca^^2+^ ^11,30,40. This property is a key aspect for the interaction of Ca^^2+^^, CaM, and their targets, but has been largely neglected by other computational models that simulate Ca^^2+^^/CaM-dependent proteins^49^.

Stimulations of our model with simple and complex patterns of Ca^^2+^^ signals resulted in formation of (Ca^^2+^^)_4_CaM with a constant Ca^^2+^^ requirement independent of the dynamic properties of these signals. Thus, in the situations tested, the interactions of Ca^^2+^^ and CaM were in fast equilibrium, which is consistent with experimental data^39^.

Our results demonstrated that AC and CaN decode the dynamic features of the Ca^^2+^^ transients because their interactions with Ca^^2+^^ occurred under kinetic control for most of the simulated protocols. Consistently with experimental observations^44^, CaN activation in our simulations increased with the number of Ca^^2+^^ signals induced by glutamate pulses, but had poor sensitivity to the frequencies of stimulation tested. In our work, the ability of CaN to count pulses was caused from a combination of its slow interaction with Ca^^2+^^ and high affinity for Ca^^2+^^/CaM, which allowed it to respond gradually and consistently to the signals used. Previous models of CaN have indicated that the high affinity of CaN for Ca^^2+^^/CaM is important for the integration of different patterns of signals^43^,^50^,^51^.

The role of AC during LTP and LTD is not completely clear^52^. Our results indicated that AC activation is less pronounced during low frequency patterns of stimulation especially at resting V_m_. Thus, AC is probably less activated during protocols of LTD than during protocols of LTP induction.

In contrast to AC, PDE1 responded to the amplitude of the Ca^^2+^^ transients independently of their dynamic properties for the large majority of the situations simulated. The regulation of PDE1 by the amplitude of the Ca^^2+^^ signals for the protocols tested indicates that it can have lower Ca^^2+^^ requirements than AC when both enzymes are stimulated by brief Ca^^2+^^ transients, which is contrary to what would be predicted from dose-response curves obtained under steady state.

In conclusion, our results demonstrated that Ca^^2+^^-dependent proteins can respond differently to the dynamic features of the Ca^^2+^^ signals to ensure the occurrence of the appropriate cellular response. The ability to decode the dynamic properties of the Ca^^2+^^ signals are caused by a combinations of factors, including differences in affinities and competing rates of reactions and mechanisms of activations, which allows some molecules to interact with Ca^^2+^^ under thermodynamic control, while others interact with it under kinetic control. Therefore, the balance between thermodynamic and kinetic control can be an important tool for decoding physiological Ca^^2+^^ signals.

## Methods

### Model Description

The computational model described in this paper was constructed using BioNetGen^53^, a rule-based software for modelling biochemical networks. The model was implemented stochastically using the SSA algorithm. The components of the model were simulated using parameters and reactions based on published experimental data. A well-mixed compartment model of 0.18 10^−15^ L was set to simulate a single hippocampal spine^54^. The model contained the synaptic NMDARs, the signalling molecules analysed (CaM, AC, PDE1 and CaN), and detailed postsynaptic mechanisms of Ca^^2+^^ dynamics (Ca^^2+^^ influx through NMDARs, buffering and extrusion) that were used to simulate physiological signals of Ca^2+^. In addition, in some simulations of the model, we included the production and degradation of cAMP by AC and PDE1, respectively, and the subsequent activation of PKA. All components of the model were validated based on comparisons with published experimental data, as described in the Results. The detailed descriptions of the reactions used to simulate each component of the model with their respective parameters and references are described in the Supplementary Information.

## Additional Information

**How to cite this article**: Antunes, G. *et al*. Modelling intracellular competition for calcium: kinetic and thermodynamic control of different molecular modes of signal decoding. *Sci. Rep.* **6**, 23730; doi: 10.1038/srep23730 (2016).

## Supplementary Material

Supplementary Information

Supplementary Dataset
