# Supplementary material for: Modelling intracellular competition for calcium: kinetic and thermodynamic control of different molecular modes of signal decoding
Source: Sci Rep. 2016 Apr 1;6:23730. doi: 10.1038/srep23730 (PMC4817061; doi:10.1038/srep23730)
Supplement: Supplementary Information [file srep23730-s1.pdf]

Modelling intracellular competition for calcium: kinetic and thermodynamic control of different molecular modes of signal decoding

Gabriela Antunes[1*], Antonio C. Roque[1], and Fabio Marques Simoes de Souza[2]

[1]Laboratory of Neural Systems (SisNe), Department of Physics, Faculdade de Filosofia Ciências e Letras de Ribeirão Preto, Universidade de São Paulo, Ribeirão Preto, Brazil

[2]Center for Mathematics, Computation and Cognition, Federal University of ABC, São Bernardo do Campo, Brazil

[*]gabrian@usp.br

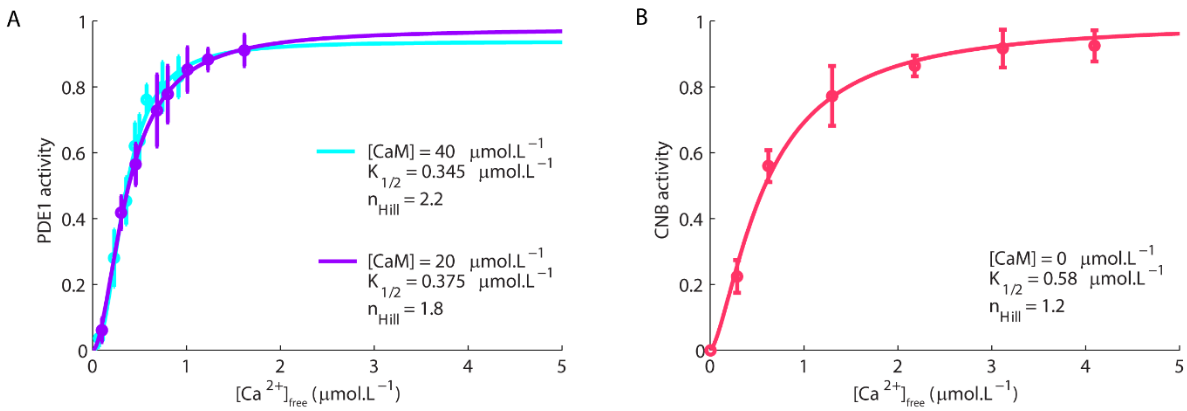

Supplementary Figure S1: Additional dose-response curves. (A) Dose-response curve of PDE1 activation as a function of the free calcium ion concentration ($[Ca^{2+}]_{free}$) in the presence of other concentrations of CaM to demonstrate that, when the concentration of CaM is saturating, the $K_{1/2}$ and n_{Hill} of the curves should not present large variations. (B) Dose-response curve of the interaction of the subunit CNB of CaN with Ca^{2+} in the absence of CaM. Experimentally, the $K_{1/2}$ for this interaction was estimated in 0.67 μmol.L^{-1}, and the nHill in 1.2 [1]. Each dot shows the average result of 10 runs of the model ± sem. The curves were fitted with 95% of confidence interval.

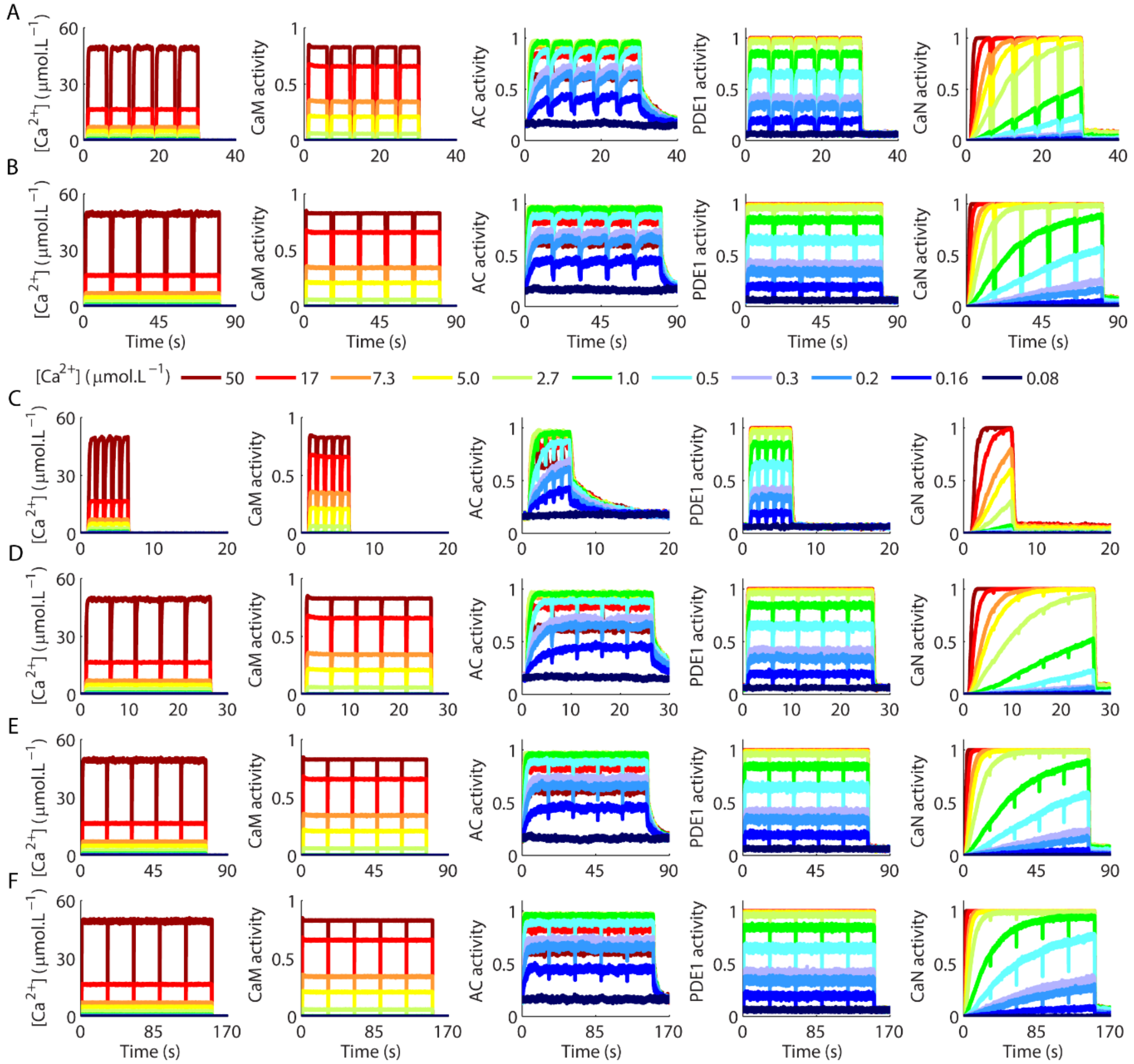

Supplementary Figure S2: Activations of the components of the model evoked by Ca^{2+} pulses. (A and B) Activation of the components of the model stimulated by five Ca^{2+} pulses with 1 s of inter pulse interval, 5 s (A) and 15 s (B) of duration and different peak concentrations. (C-F) Average responses of the components of the model to five Ca^{2+} pulses released with 100 ms of inter pulse interval, durations of 1 s (C), 5 s (D), 15 s (E), and 30 s (F) and different peak concentrations. Each curve is the average of 10 runs of the model. The maximum activity of AC, CaN, and PDE1 corresponded to 1 μmol.L^{-1}. The maximum activity of CaM corresponded to 40 μmol.L^{-1}. The term CaM refers to CaM loaded with Ca^{2+}.

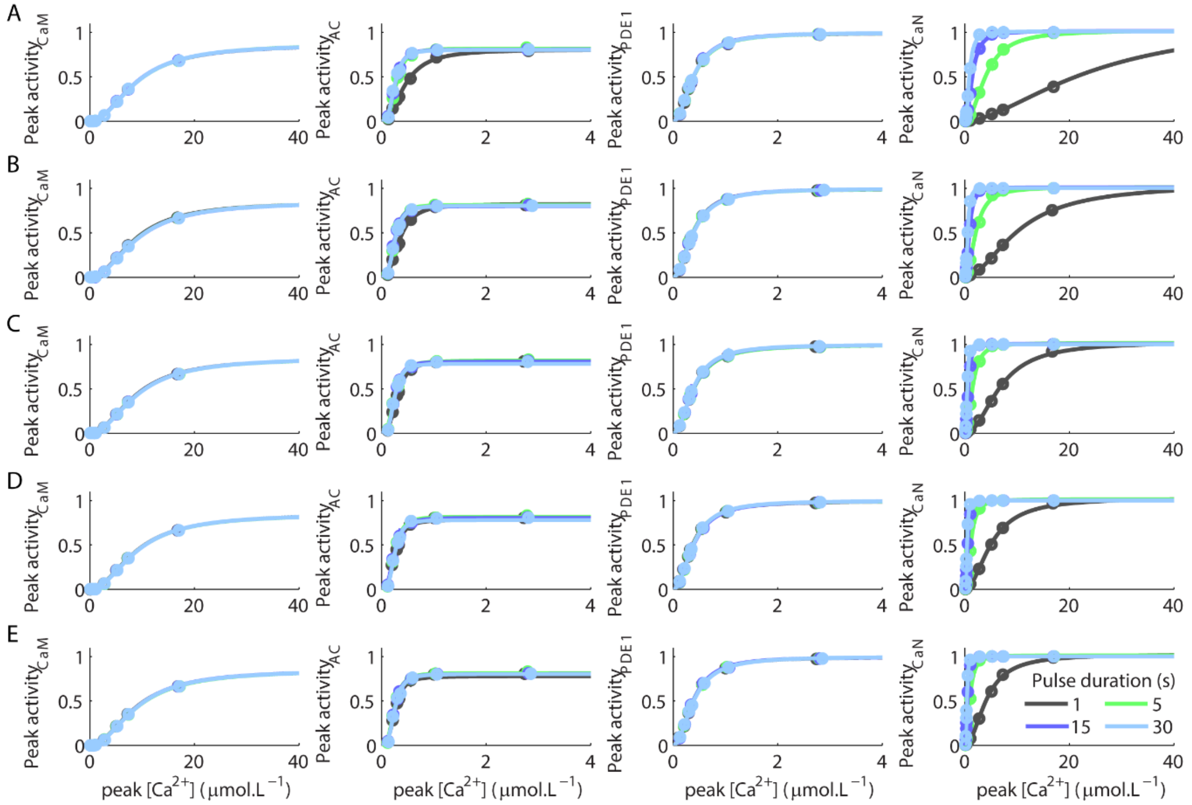

Supplementary Figure S3: Sigmoid curves of the activation of CaM, AC, PDE1 and CaN as a function of the peak amplitudes of Ca^{2+} transients. Trains of five Ca^{2+} pulses released with 100 ms of inter pulse interval were used to evoke the activation of CaM, AC, PDE1 and CaN (Fig. S3 C-F). Each train consisted of five Ca^{2+} pulses with a specific duration and peak amplitude. The duration of the pulses varied from 1 to 30 s, and their peak amplitude from 0.08 μmol.L^{-1} to approximately 50 μmol.L^{-1}. The peak activation of CaM, AC, PDE1 and CaN obtained as a function of the peak concentration of each one of the five Ca^{2+} pulses in a given train was used to plot dose-response curves. Panels A-E show the dose-response curves obtained for the first (A), the second (B), the third (C), the fourth (D), and the fifth (E) Ca^{2+} pulse of each train. These results indicated that the number of Ca^{2+} pulses and their durations regulated the activation of CaN and AC, but had no effects on the activation of CaM and PDE1.

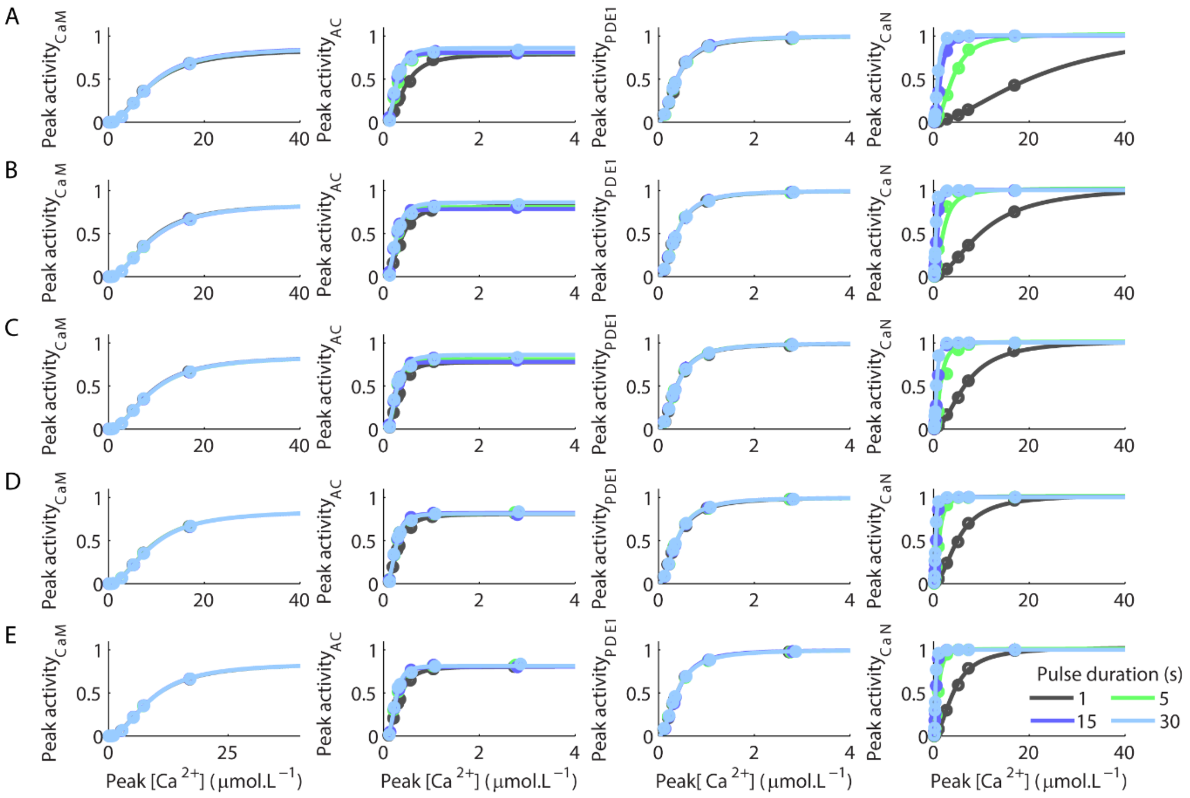

Supplementary Figure S4: Sigmoid curves of the activation of CaM, AC, PDE1 and CaN as a function of the peak amplitudes of Ca^{2+} transients. Trains of five Ca^{2+} pulses released with 1 s of inter pulse interval were used to evoke the activation of CaM, AC, PDE1 and CaN as showed in Fig. 2A-B and Supplementary Fig. S2 A-B. Each train consisted of five Ca^{2+} pulses with a specific duration and peak amplitude. The duration of the pulses varied from 1 to 30 s, and their peak amplitude from 0.08 μmol.L^{-1} to approximately 50 μmol.L^{-1}. The peak activation of CaM, AC, PDE and CaN obtained as a function of the peak concentration of each one of the five Ca^{2+} pulses in a given train was used to plot dose-response curves. Panels A-E show the dose-response curves obtained for the first (A, the same results appear in Fig. 2C-F), the second (B), the third (C), the fourth (D), and the fifth (E) Ca^{2+} pulse of each train.

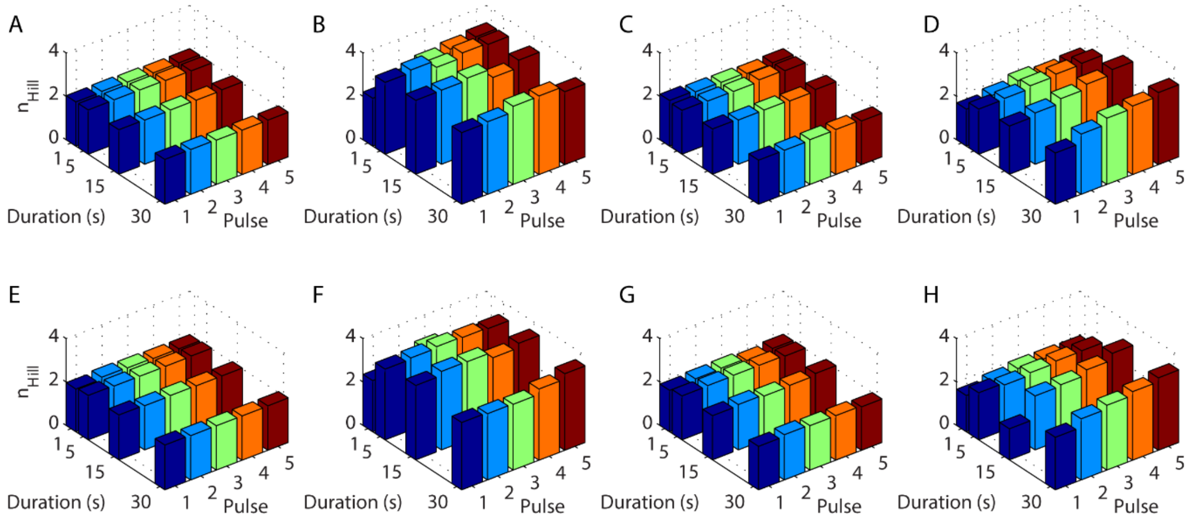

Supplementary Figure S5: Values of n_{Hill} obtained for each sigmoid curve showed in Fig. 2C-F and Supplementary Fig. S3-S4. The results indicate little variation of n_{Hill} as functions of the number of pulses used to stimulate the model, the durations of the pulses, and their inter pulse interval. A-D: n_{Hill} of CaM (A), AC (B), PDE1 (C), and CaN (D) obtained for pulses released with 1 s of inter pulse interval. E-H: n_{Hill} of CaM (E), AC (F), PDE1 (G), and CaN (H) obtained for pulses released with 100 ms of inter pulse interval.

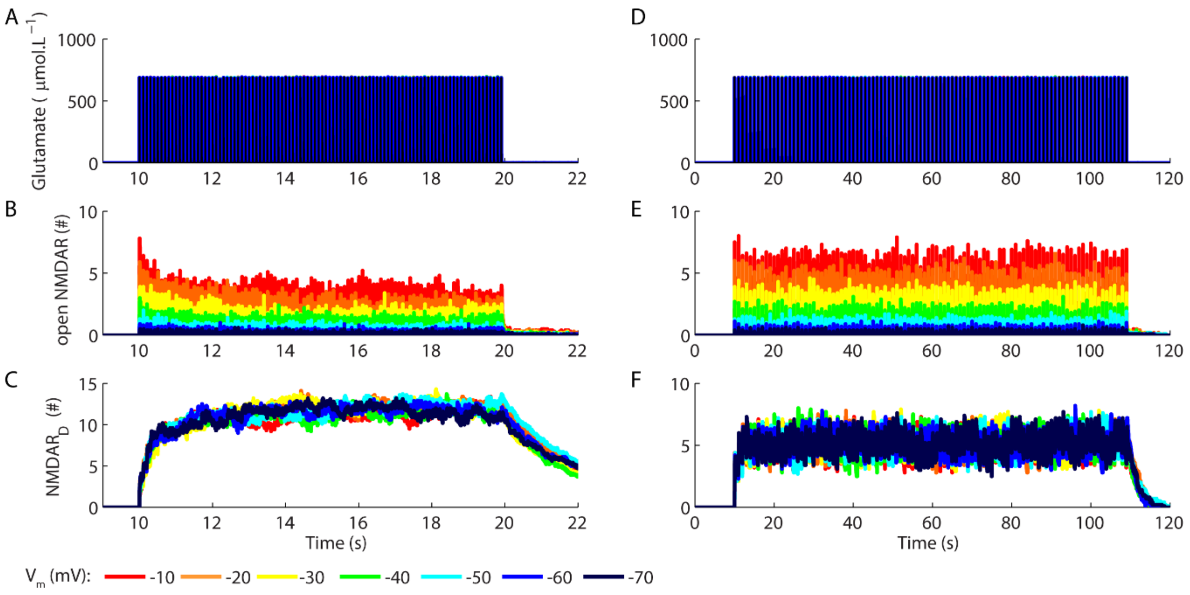

Supplementary Figure S6: Number of open NMDARs and desensitized NMDARs ($NMDAR_D$) evoked by 100 pulses of glutamate released at 10 Hz and 1 Hz. Time courses of open NMDARs and desensitized NMDARs ($NMDAR_D$) evoked by 100 pulses of glutamate released at 10 Hz (A-C) and 1 Hz (D-F) with V_m varying from -10 mV to -70 mV. Each curve shows the mean result of 10 runs of the model.

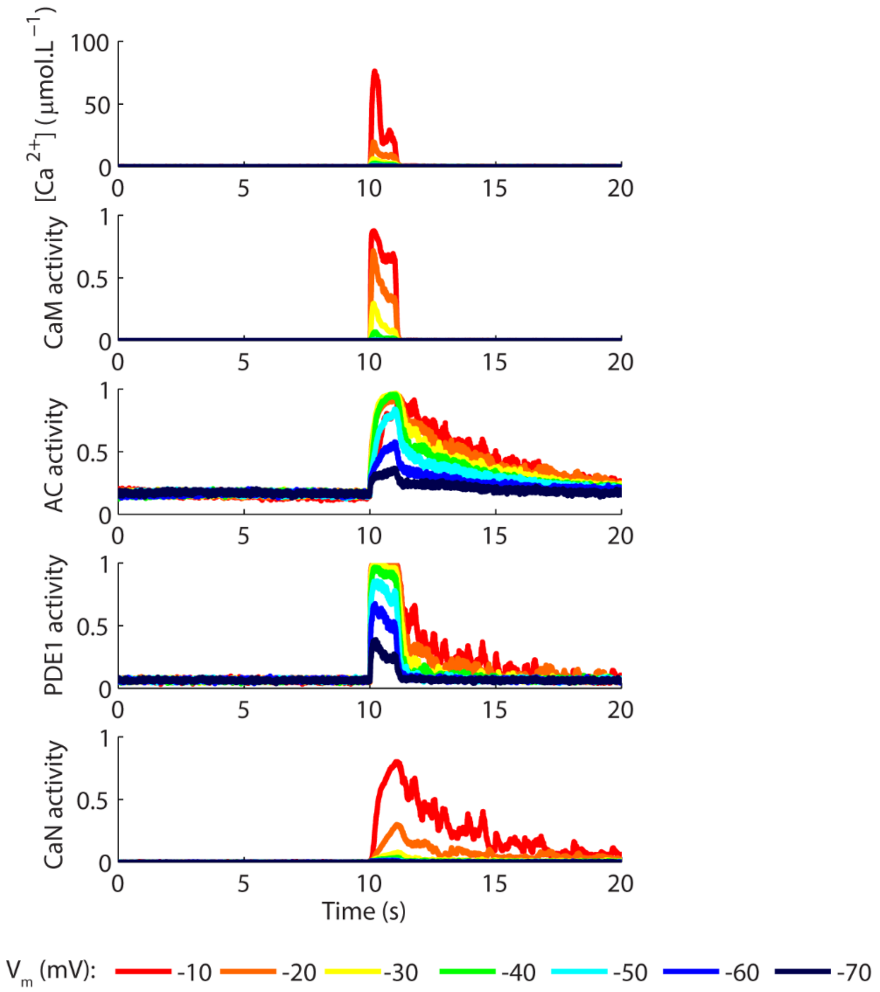

Supplementary Figure S7: Stimulation of the model with 100 glutamate pulses released at 100 Hz. Each curve shows the mean result of 10 runs of the model.

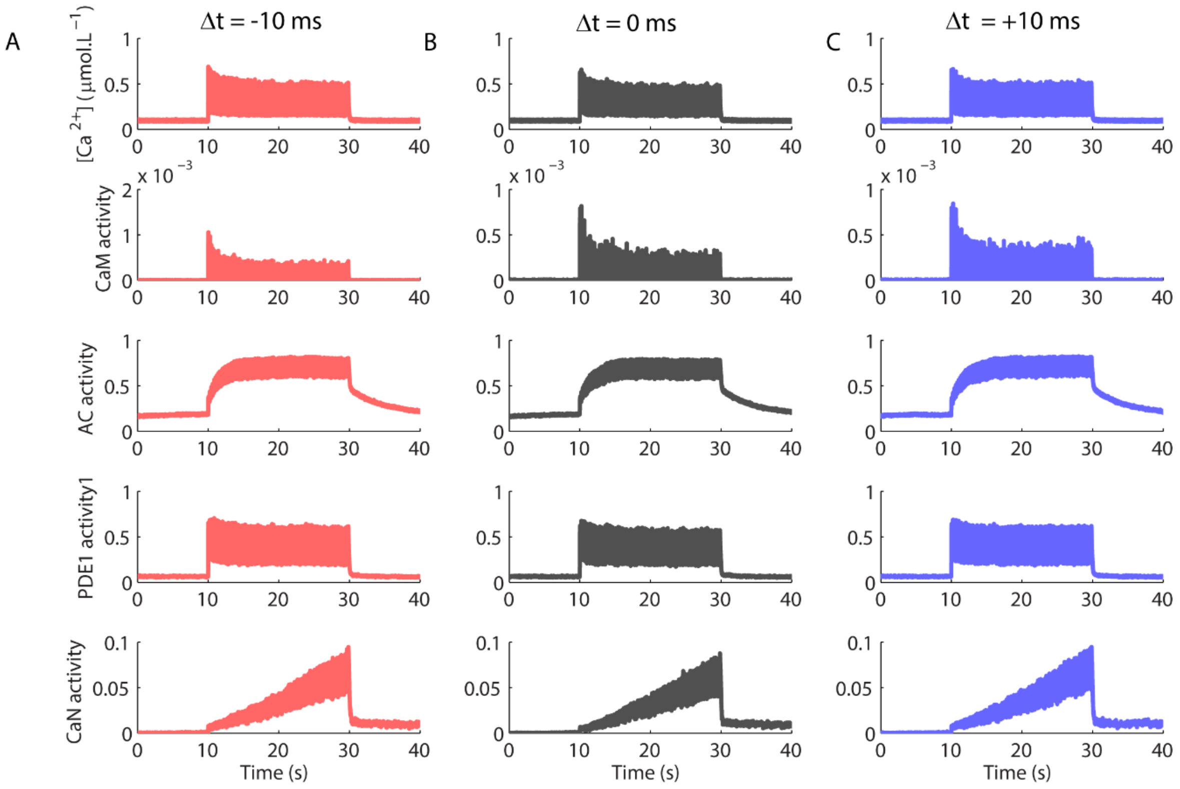

Supplementary Figure S8: Activation of the components of the model during protocols of STDP. The protocols consisted of sixty pulses of glutamate (amplitude of 700 μmol.L^{-1}, 1 ms of duration) at 5 Hz paired with postsynaptic spikes. Each glutamate pulse was paired with a burst of three action potentials at 50 Hz [2,3]. According to experimental evidences, changes in the time interval (Δt) between the glutamate pulse and the burst of postsynaptic spikes is enough to promote the occurrence of opposite forms of synaptic plasticity [4]. The spikes were simulated occurring 10 ms prior to each glutamate pulse (Δt = -10 ms) (A), simultaneously with the glutamate pulses (Δt = 0 ms) (B), or10 ms after each glutamate pulse (Δt = +10 ms) (C). Δt was defined by the closest action potential in time to the onset of the glutamate pulse. In our system, alterations of Δt did not promote significant differences in the Ca^{2+} signals of the model, which is consistent with evidences that STDP requires other sources of Ca^{2+} including voltage-dependent Ca^{2+} channels and metabotropic glutamate

receptors [3]. AC, PDE1 and CaN responded differently to the Ca^{2+} signals, but they did not discriminate Δt. Each curve is the average result of 50 runs of the model.

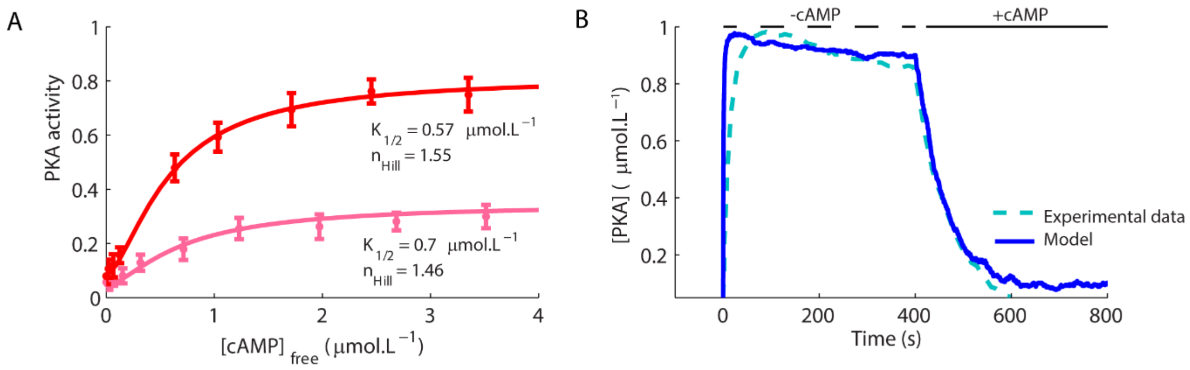

Supplementary Figure S9: Validation of the reactions and parameters used to simulate PKA. (A) Dose-responses curves of PKA activation as a function of the free concentration of cAMP ([cAMP]$_{free}$) in presence or absence of an excess of its substrate Kemptide (1000 µmol.L^{-1} of Kemptide and 1 µmol.L^{-1} of PKA), which regulates its activation[5]. The values of $K_{1/2}$ and n_{Hill} are in accordance with published experimental data ($K_{1/2}$ varying around 0.58-0.6 µmol.L^{-1} and n_{Hill} of 1.7 [6,7]). (B) Time course for the association/dissociation of the regulatory (R) and catalytic (C) subunits of PKA in absence and presence of cAMP (500 µmol.L^{-1}) obtained with the model in comparison to an experimental curve from the literature[8]. See Supplementary Information (*cAMP dynamics and PKA*) for further details.

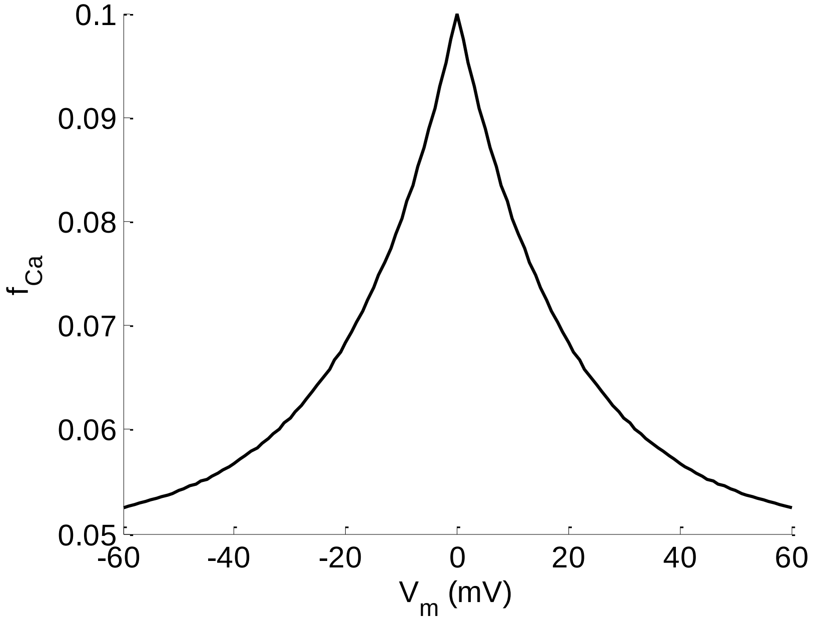

Supplementary Figure S10: The fractional calcium (f_{Ca}) versus the membrane potential (V_m) calculated with equations 2 and 3.

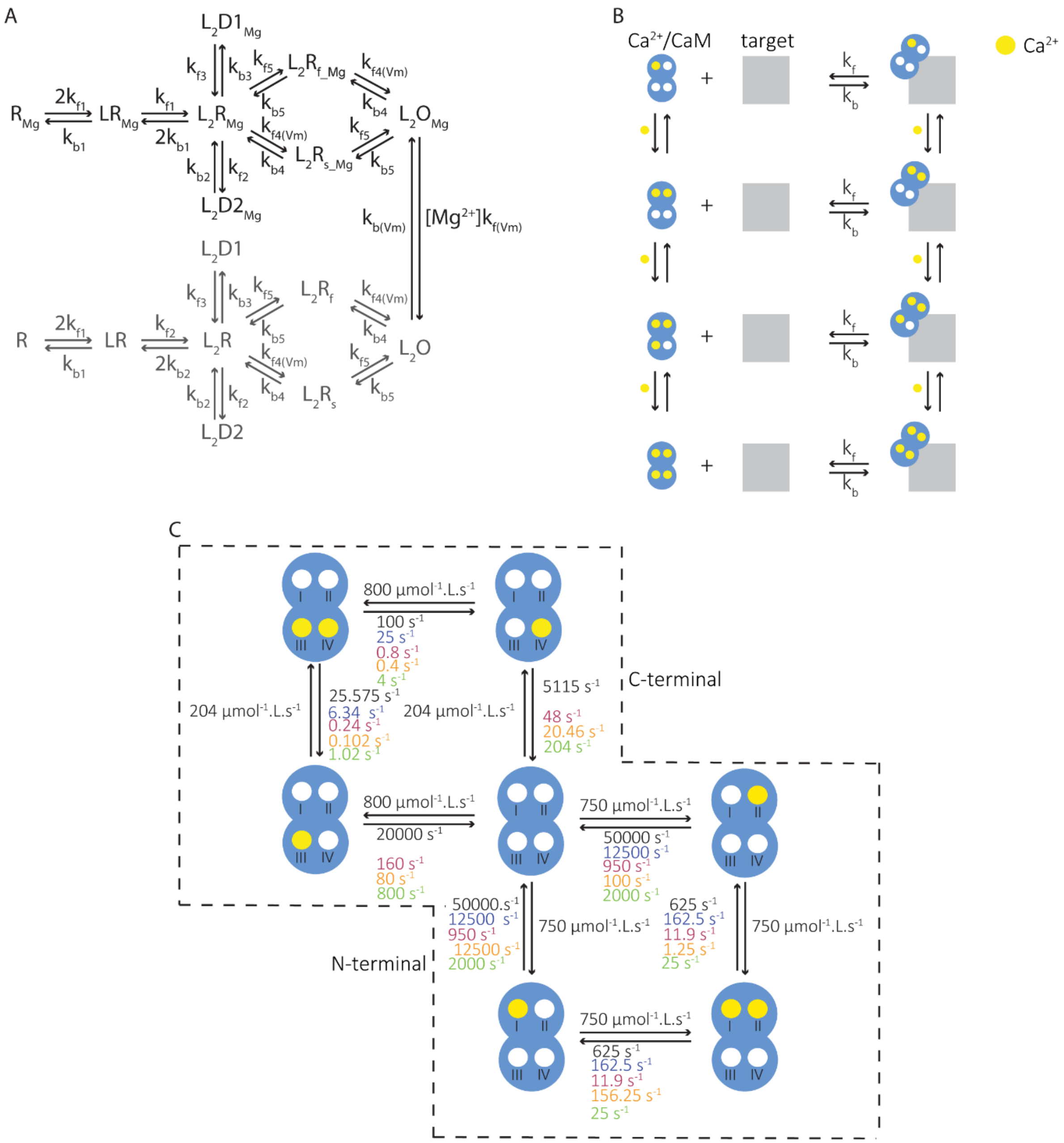

Supplementary Figure S11: Schematic representations of the components of the model and their mechanisms of interactions. (A) Schematic representation of the kinetic model used to simulate the NMDARs (R: NMDAR; L: glutamate; R_{Mg}: blocked NMDAR; R_s and R_f: intermediary states of the receptors; D1 and D2: desensitized receptors, O: open receptor; V_m: membrane potential; see references[9–11] for details). (B) Schematic representation of the

interaction between Ca^{2+}/CaM and its targets. For all targets simulated, the binding to Ca^{2+}/CaM com occur with one, two, three of four ions associated to its structure. For the interaction between PMCA and Ca^{2+}/CaM, CaM must be associated to one or two ions in the Ca^{2+}-binding sites located at the C-terminal. (C) The interaction of Ca^{2+} and the four Ca^{2+}-binding sites of CaM. The different Ca^{2+}-binding sites of CaM (I, II, III and IV) are represented in the cartoon. In the presence of its targets, CaM has a higher affinity for Ca^{2+}, which was simulated through changes in the rate constants for the dissociation of Ca^{2+} to each Ca^{2+}-binding site (rate constants for the dissociation of Ca^{2+} from CaM in the absence of targets (black), in presence of PMCA (blue), CaN (magenta), AC (orange), and PDE1 (green)).

Supplementary Methods

Descriptions of the reactions and parameters used to simulate each component of the model. The model was solved stochastically using the algorithm Gillespie SSA.

NMDARs

NMDARs are glutamatergic ionotropic receptors that mediate the influx of Ca^{2+} to the cytosol of the hippocampal spine during the induction of long-term forms of synaptic plasticity[12,13]. Structurally, hippocampal neuronal NMDARs are composed by two GluN2 subunits, which are the subunits that bind glutamate, combined with two GluN1 subunits that bind to the co-agonists L-glycine and D-serine[14]. For simplicity, we assumed that NMDAR co-agonists are constantly present in the synaptic cleft, which is justified by the continuous release and uptake of L-glycine and D-serine from the extracellular environment [14]. Therefore, they were not simulated explicitly and the gating of NMDARs in the computational model was controlled solely by the glutamate concentration. According to the native composition of synaptic NMDARs[12], two types of receptors with distinct kinetic properties were included in the model, di-heteromerics receptors GluN1/GluN2A, which account for approximately 65% of the NMDARs in the hippocampal CA1 of adult animals, and the receptors GluN1/GluN2B that was considered to represent the remaining 35% of the total NMDARs [15,16]. A total of 20 NMDARs were included in the model[17]. Both receptor types were simulated with the same set of reactions, but using different rate constants for the state transitions[11].

A unique feature of NMDARs is their mechanism of activation that requires both the binding of glutamate and the depolarization of the postsynaptic cell membrane to promote the release of magnesium ions (Mg^{2+}) that block the pore of their channels in a membrane

potential (V_m)-dependent manner[12,18]. The depolarization-dependent removal of Mg^{2+} occurs only from the open channel[18]. In consequence, NMDARs detect the coincidence between the presynaptic activation, which releases glutamate, and the postsynaptic membrane depolarization[13]. The kinetic model used to simulate both types of NMDARs in this work was based on a previous model developed to simulate the gating of NMDAR channels in absence of Mg^{2+} [19], which was recently expanded to incorporate the interaction of NMDARs with Mg^{2+} (Supplementary Figure S11A)[9,10]. All the rate constants used to simulate each type of NMDAR were taken from published models[9–11]. The complete description of the reactions and parameters used to simulate the NMDARs is listed in Supplementary Table I (Reac1-Reac17).

***Ca^{2+}* Dynamics**

The mechanisms of Ca^{2+} dynamics implemented in our stochastic model consisted of Ca^{2+} influx, buffering and extrusion from the cytosol. We have not included Ca^{2+} diffusion across the spine neck to its parental dendrite in our model because it accounts for less than 10% of the Ca^{2+} clearance [20].

The influx of Ca^{2+} in the model occurs exclusively through NMDAR channels, and was given by the follow equation:

$$Influx_{NMDAR} = \frac{\sum NMDAR_{open}\, I_{NMDA} f_{Ca} n_A}{2F.Vol_{spine}} \tag{1}$$

where $NMDAR_{open}$ stands for the number of open NMDAR channels unblocked by Mg^{2+}

(Supplementary Figure S11A), I_{NMDA} is the maximum unitary channel current (5 pA)[21], F is the Faraday constant (96485 $C.mol^{-1}$), 2 refers to the Ca^{2+} valence, n_A is the Avogadro number (6.02 10^{23} mol^{-1}) and f_{Ca} is the fraction of Ca^{2+} from the I_{NMDA}.

Experimental data demonstrated that the fractional Ca^{2+} current of the NMDARs is regulated by the V_m and corresponds to approximately 10-17% of the NMDAR-mediated current [22–25]. Based on experimental observations of $f_{Ca}(V_m)$, we used an exponential description with a maximum fractional Ca^{2+} current of 10% at the reversal potential (Supplementary Fig. S10):

$$f_{Ca}(V_m < 0) = 0.05 + 0.05\ exp((V_m - E_{rev})/20) \quad (2)$$

$$f_{Ca}(V_m >= 0) = 0.05 + 0.05\ exp(-(V_m - E_{rev})/20) \quad (3)$$

where V_m is the membrane potential, E_{rev} is the NMDAR reversal potential (0 mV). However, since BioNetGen[26], the software used to implement the model, does not allow the explicit simulation of equations such as (1), we simulated the influx of Ca^{2+} in the BioNetGen as a first order reaction:

$$NMDAR_{open} \xrightarrow{k_{influx}} NMDAR_{open} + Ca^{2+}$$

The rate constant k_{influx} was estimated with a multiplicative factor (8.10^6 $mol^{-1}.L$) applied to equation (1) to obtain the correct time course and peak [Ca^{2+}]:

$$k_{influx} = 8e6\,\frac{\sum NMDAR_{open}\, I_{NMDA} f_{Ca} n_A}{2F.Vol_{spine}} \quad (4)$$

Two Ca^{2+} buffers were included in the model: an unspecific buffer (UB) and CaM, which act as a buffer and as a Ca^{2+}-signal mediator[27,28]. The interaction of Ca^{2+} to UB was simulated with the binding of a single ion to each UB molecule. Thus, UB interacts with Ca^{2+} through a reaction for the complex formation ($Ca^{2+} + UB \overset{k_f}{\rightarrow} (Ca^{2+})UB$, k_f = 50 µmol^{-1}.L.s^{-1}), and another for its dissociation ($(Ca^{2+})UB \overset{k_b}{\rightarrow} Ca^{2+} + UB$, k_b = 500 s^{-1}) (Supplementary Table I, Reac19).

In contrast to UB, CaM interacts with four Ca^{2+} that bind to two pairs of Ca^{2+} binding sites located in two distinct globular domains (the C-terminal and N-terminal domains) [27,28]. Each pair of Ca^{2+}-binding sites of CaM binds Ca^{2+} sequentially and with positive cooperativity, but there is no cooperativity among sites located on distinct CaM domains[29]. The Ca^{2+}-binding sites located at the N-terminal domain are termed I and II and the sites located at the C-terminal are termed III and IV. To simulate the interaction between CaM and Ca^{2+}, it was assumed that each CaM domain has two macroscopic association constants, K_1 and K_2. K_1 is the sum of the microscopic equilibrium constants assuming that individual Ca^{2+}-binding sites of either domain are occupied sequentially[30,31]:

$K_{N1} = k_I + k_{II}$, for the N-terminal domain (5)

$K_{C1} = k_{III} + k_{IV}$, for the C-terminal domain (6)

where k_I, k_{II}, k_{III}, and k_{IV}, are the microscopic equilibrium constants of Ca^{2+}-binding sites I,

II, III, and IV respectively. The cooperativity observed for the binding of a second Ca^{2+} to either CaM globular domain defines that[30,31]:

$$K_{N2} = k_n k_I k_{II}, \text{ for the N-terminal domain} \quad (7)$$

$$K_{C2} = k_c k_{III} k_{IV}, \text{ for the C-terminal domain} \quad (8)$$

where k_n and k_c are the intradomain cooperative constants for the binding of the second Ca^{2+} to the N- and C-terminal, respectively [30,31]. A complete description of the reactions and rate constants used in the model was published previously [32] and is listed in the Supplementary Table I (Reac20- Reac27).

The concentration of both buffers, CaM and UB, were set as 40 µmol.L^{-1} in order to reproduce the endogenous buffer capacity (k_E) observed in the CA1 dendritic spines. The k_E is defined, in generic terms, as the ratio between the Ca^{2+} ions bound to buffers and the number of ions that are free. Taking the mean number of free Ca^{2+} during simulated resting conditions (~ 6) and the number of bound Ca^{2+} (~ 18 ions bound to the UB and 100 ions bound to CaM), the value of k_E obtained with the model is approximately 20 as observed in native hippocampal spines [20].

Two different species account for Ca^{2+} extrusion in the model: the plasma membrane Ca^{2+}-ATPase (PMCA) and the Sodium (Na^+)/Ca^{2+}-exchanger (NCX). PMCA is activated by binding to Ca^{2+}/CaM, which promotes an increase in its catalytic rates through the release of an autoinhibitory domain close to its catalytic site, and a drop of its K_D for Ca^{2+} from 10-30 µmol.L^{-1} to 0.2-0.75 µmol.L^{-1} [33]. To simulate the activity of PMCA, a basal catalytic rate was implemented representing the activity of the pump in absence of saturating Ca^{2+}/CaM [33]. The basal catalytic activity was simulated considering the reversible formation of a complex

between the PMCA and Ca^{2+} using one reaction to simulate the complex formation ($Ca^{2+} + PMCA \overset{k_f}{\rightarrow} (Ca^{2+})PMCA$), k_f = 55 µmol^{-1}.L.s^{-1}), another for the complex dissociation ($(Ca^{2+})PMCA) \overset{k_b}{\rightarrow} Ca^{2+} + PMCA$, k_b = 250 s^{-1}), and an irreversible step that represents the pumping of Ca^{2+} to the outside of the cellular compartment ($(Ca^{2+})PMCA) \xrightarrow{k_{cat}} PMCA$, k_{cat} = 30 s^{-1}) [33,34]. Note that the Ca^{2+} in the outside of the cellular compartment was not simulated explicitly.

To simulate the interaction of PMCA to CaM, we assumed that it can occur at low [Ca^{2+}] involving partially loaded forms of the complex Ca^{2+}/CaM (Supplementary Figure S11B) [35]. Such interaction does not lead to the release of the autoinhibitory domain of PMCA, but are likely to contribute to a fast activation of PMCA when the level of Ca^{2+} rises [35]. However, we assumed that the binding of Ca^{2+}/CaM to PMCA is sequentially ordered: the C-terminal associates first, followed by the subsequent binding of the N-terminal[36,37]. Thus, we assumed that CaM associated with one or two Ca^{2+} at the C-terminal can interact with PMCA. The binding of both CaM terminals are required for full activation of PMCA[38]. The association of CaM to PMCA increases its affinity for Ca^{2+} as observed for other CaM targets. A four-fold increase in the microscopic equilibrium constants for the binding of Ca^{2+} to CaM associated with PMCA was used in the model, which was implemented through a four-fold reduction in the rate constants for the dissociation of Ca^{2+} from CaM bound to PMCA based on experimental data (Supplementary Figure S11C, Supplementary Table I: Reac28-Reac38) [34,39].

A second species of the model that regulates the intracellular levels of [Ca^{2+}] is NCX, which is a bidirectional transporter that exchange Na^+/Ca^{2+}, but in the model the transport of Na^+ were not implemented explicitly. The efflux of Ca^{2+} was simulated as a non-conservative

process, and extracellular [Ca^{2+}] was not modelled explicitly as described previously [32] (Supplementary Table I: Reac39). A constant first-order rate of leak of Ca^{2+} was included in the model to counteract the basal activity of PMCA and NCX and to maintain the resting [Ca^{2+}] around 80-150 nmol.L^{-1} (Supplementary Table I: Reac40).

CaN

CaN is a heterodimeric enzyme composed by a catalytic subunit (CNA), which contains a Ca^{2+}/CaM-binding site, and a regulatory subunit (CNB) with four Ca^{2+}-binding sites, two of them with very high affinities for Ca^{2+} and two with moderate affinities [40,41]. The interaction of Ca^{2+} with CNB is a precondition for the binding of Ca^{2+}/CaM to CNA[1,40,42,43].

Structurally, CNB is very similar to CaM and consists of two globular domains, each one containing a pair of Ca^{2+}-binding sites [44]. Based on this similarity, we used the same approach implemented to simulate the interaction of Ca^{2+} with CaM to define the microscopic parameters for the interaction of Ca^{2+} to CNB. Thus, we considered that the binding of Ca^{2+} to each CNB globular domain has two macroscopic association constants, K_1 and K_2. K_1 is the sum of the microscopic equilibrium constants for the binding of the first ion to either pair of Ca^{2+}-binding sites (equations (5) and (6)) assuming that the sites are occupied sequentially[30,31]. K_2 is the macroscopic constant for the binding of the second ion to either pair of Ca^{2+}-binding sites, and it is defined by equations (7) and (8) [30,31]. The Ca^{2+}-binding sites located in the CNB C-terminal, termed EF-hand III and IV, have higher Ca^{2+} affinities than the EF-hands I and II, located at the N-terminal[5]. The macroscopic binding constants for these sites have been determined: 0.094 μmol.L^{-1} (K_{C1}), 0.036 μmol.L^{-1} (K_{C2}), 1.1 μmol.L^{-1} (K_{N1}), and 0.6 μmol.L^{-1} (K_{N2})[5,41]. Assuming that the microscopic affinity for either site of

a given CaN domain is equivalent, we calculated the values of k_{III} and k_{IV} (0.047 µmol.L^{-1}), k_I and k_{II} (0.55 µmol.L^{-1}), and the values of the cooperative constant for the C-terminal (k_c = 16.29 µmol^{-1}.L), and for the N-terminal (k_n = 2 µmol^{-1}.L). The backward rate constant (k_b) for the unbinding of the first Ca^{2+} from the C-terminal was set as 0.03 s^{-1} [33], which resulted in a forward rate constant (k_f) of 0.64 µmol^{-1}.L.s^{-1}. The value of k_f was kept equal for the binding of Ca^{2+} to both sites on the same terminal. In consequence, the k_b for the dissociation of Ca^{2+} from the second site filled was calculated as 0.0018 s^{-1}. For the sites located at the N-terminal, assuming a k_b of 0.05 s^{-1} [41], the k_f for the binding of the first Ca^{2+} was calculated as 0.09 µmol^{-1}L.s^{-1}. Then, the k_b for the dissociation of the second Ca^{2+} from the N-terminal was calculated as 0.025 s^{-1}. The bindings of Ca^{2+} to the Ca^{2+}-binding sites located on different domains of CNB were simulated as independent events (Supplementary Table I: Reac41-Reac44).

After the binding of Ca^{2+} to CNB, CNA interacts with Ca^{2+}/CaM with a stoichiometry of 1:1 [41–43]. CaN has the highest affinity reported for CaM fully loaded with Ca^{2+} (~ pmol.L^{-1}) [45]. It can also interact with CaM partially loaded with Ca^{2+}, but with lower affinity [46]. We simulated the interaction of CaN with fully loaded Ca^{2+}/CaM using parameters reported in the literature (for the complex formation k_f = 46 µmol^{-1}.L.s^{-1}, and for the complex dissociation k_b = 0.0012 s^{-1}) [45]. To implement the interaction between CaN and CaM partially loaded with Ca^{2+}, we kept the k_f unchanged (46 µmol^{-1}.L.s^{-1}), and recalculate k_b using affinities reported for the interaction of CaN with isolated domains of CaM (K_D = 1 and 7 µmol.L^{-1} for the C (k_b = 46 s^{-1}) and N-terminal (k_b = 322 s^{-1}), respectively) [46] (Supplementary Table I: Reac45-Reac48). Like many other CaM targets[47–49], CaN increases considerable the affinity of Ca^{2+} for CaM but it preserves the order of magnitude that separates the affinities between the Ca^{2+}-binding sites located at the C and N-terminal [46]. This process was

implemented using equations (5)-(8) to calculate the rate constants. The values of k_f for the interaction between Ca^{2+} and the Ca^{2+}-binding sites of CaM in the presence or absence of CaN were kept unchanged because the presence of CaM targets usually modifies the rate constants for the Ca^{2+} dissociation from CaM structure [47,48]. The k_bs for the interaction of the first and second ion to each CaM domain associated to CaN were calculated considering the microscopic binding for the sites k_I and k_{II} (0.8 μmol^{-1}.L) and k_{III} and k_{IV} (5 μmol^{-1}.L). We kept k_n (80 μmol^{-1}.L), and k_c (200 μmol^{-1}.L) unchanged by the presence of CaN [32]. Thus, the values for the dissociation rate constants were: k_{b_I} and k_{b_II} = 950 (11.9) s^{-1}, k_{b_III} = 160 (0.8) s^{-1}, and k_{b_IV} = 48 (0.24) s^{-1} (the number in parenthesis are the rate constants for the dissociation of Ca^{2+} when both Ca^{2+}-binding sites on a given domain are filled) (Supplementary Figure S11C, Supplementary Table I: Reac48-Reac55).

AC

In our model, AC activity was regulated solely by its interaction with Ca^{2+}/CaM. Note, however, that its activity can also be stimulated by the subunit $G_{s\alpha}$ of the trimeric G protein, and by forskolin (except AC9), a plant diterpene [50]. AC can interact with CaM fully loaded or partially loaded with Ca^{2+} [51]. The stimulation of AC by partially loaded CaM requires, preferentially, the filing of at least two Ca^{2+}-binding sites of CaM (sites II and IV), which implies that AC must interact with both CaM lobes simultaneously [52]. Assuming that the affinity between CaM and AC is equal to the concentration of CaM required to activate the half-maximum concentration of AC available, the absence of Ca^{2+} bound to site II or site IV of CaM promotes a 15-fold and 30-fold decrease in the affinity of AC for CaM, respectively [51]. The filling of the Ca^{2+}-binding site III is also important and its absence promotes a 5-fold reduction in the affinity of the complex formed [51]. However, the filling of

site I causes only a 2-fold reduction in the affinity of AC for CaM [51]. Thus, we implemented the interaction of AC partially loaded with Ca^{2+} considering that the absence of the filling of site IV, II, III, and I promotes a 30-fold, 15-fold, 5-fold, and a 2-fold decrease in AC affinity for CaM, respectively [51]. The absence of Ca^{2+} bound to any two or three binding sites simultaneously was simulated considering a multiplicative effect in the decrease of the affinity for the interaction between AC and CaM. Thus, for example, the absence of Ca^{2+} bound to sites II and III, or sites III and IV, or sites II and IV, or sites I, II and IV was simulated with an affinity 75-fold, 150-fold, 450-fold and 900-fold smaller than the affinity of AC for CaM fully loaded with Ca^{2+}. The affinity for the interaction between CaM fully loaded with Ca^{2+} and AC was defined as the EC_{50} measured experimentally (0.05 $\mu mol.L^{-1}$)[51]. The rate constant for the dissociation between AC and Ca^{2+}/CaM was estimated based on the half-time for the dissociation of CaM from synaptic membranes ($k_b = 0.01\ s^{-1}$)[53]. With these values, the rate constant for the association of AC with CaM fully loaded with Ca^{2+} was calculated ($k_f = 0.2\ \mu mol^{-1}.L.s^{-1}$). To simulate the interaction between AC and the other states of CaM partially loaded with Ca^{2+}, we kept k_f unchanged and recalculated k_b considering the decrease in the affinity between AC1 and CaM described above (Supplementary Table I: Reac56-Reac70).

Experimental data have indicated that the presence of AC greatly decreases the rate for Ca^{2+} dissociation from CaM for three sites, which we assumed to be sites II, III, and IV, but appears to play a less significant role for a forth site (site I)[52]. Thus, we kept the rate constants for the association of Ca^{2+} to CaM in presence or absence of AC unchanged. We considered a 500-fold decrease in the rate constant for the dissociation of Ca^{2+} from site II, a 250-fold decrease in the dissociation of Ca^{2+} from site III or IV, and a 4-fold reduction in the dissociation of Ca^{2+} from Ca^{2+}-binding site I of CaM in presence of AC (Supplementary

Figure S11C, Supplementary Table I: Reac71-Reac78). These values were based on experimental data[52], but were adjusted to ensure that the model of AC was able to simulate the Ca^{2+} requirement observed experimentally, measured as the $K_{1/2}$ (Fig. 1I).

Not only Ca^{2+} stimulates AC through the complex Ca^{2+}/CaM, but it also inhibits its activity at high concentrations (~50-80 μM) *in vitro* and *in vivo*[54,55]. Ca^{2+} inhibition of AC was implemented as simple reactions of binding/unbinding ($Ca^{2+} + AC \overset{k_f}{\rightarrow} (Ca^{2+})AC$, k_f = 0.1 $\mu M^{-1}.s^{-1}$, $(Ca^{2+})AC \overset{k_b}{\rightarrow} Ca^{2+} + AC$, k_b = 8 s^{-1}), and the parameters used were estimated from the K_D observed experimentally[55] (Supplementary Table I: Reac79). We assumed that the inhibitory binding of Ca^{2+} to AC is independent of AC interaction with the Ca^{2+}/CaM complex.

PDE1

Eleven subfamilies of PDEs have been identified and, in all organisms, multiple isoforms of PDEs appear to be involved in the tight control of cAMP concentration[56]. PDE1A2, a member of the PDE1 subfamily, is highly expressed in the brain[57]. PDE1A2 (referred here simply as PDE1) can interact with CaM fully or partially saturated with Ca^{2+}, but it requires CaM bound to three or four ions to be stimulated[58,59]. CaM fully saturated with Ca^{2+} interacts with PDE1 with affinity (K_D) of 1 $nmol.L^{-1}$ [57], and a Ca^{2+} requirement of approximately 300 $nmol.L^{-1}$ [60]. The interaction between PDE1 and Ca^{2+}/CaM was simulated with simple reactions of association/dissociation (Supplementary Figure S11B). The rate constants for these reactions were estimated from the K_D and based on a slow half-life for the dissociation reaction, which was used to estimate a rate constant of dissociation of (k_b) 10^{-3} s^{-1} [59]. Consequently, the rate constant for the association of PDE1 with CaM fully

saturated with Ca^{2+} was set as 1 $\mu mol^{-1}.L.s^{-1}$. This rate was kept unchanged for the interaction between PDE1 and CaM partially loaded with Ca^{2+}. To simulate the interaction between PDE1 and CaM partially loaded with Ca^{2+}, we assumed that the absence of Ca^{2+} bound to the Ca^{2+}-binding sites located at the N-domain of CaM have a more pronounced effect in the affinity between PDE1 and Ca^{2+}/CaM [54]. Thus, for the interaction of PDE1 with CaM partially loaded with three ions and one vacant Ca^{2+}-binding sites at CaM C-terminal, we assumed no change in the affinity between PDE1 and Ca^{2+}/CaM [54]. However, we considered a 10-fold decrease in the affinity of the resulting complex PDE/Ca^{2+}/CaM if a single Ca^{2+} was absent from one of the sites located at CaM N-terminal [61], which was simulated by an increase in the rate for the complex dissociation ($k_b = 10^{-2}$ s^{-1}). To simulate the interaction between PDE1 and partially loaded CaM with 2 ions absent from its C-terminal, we assumed an increase of 50-fold (50-fold reduction in the affinity) in the rate of dissociation of the complex PDE/Ca^{2+}/CaM ($k_b = 0.5$ s^{-1}). For the absence of 2 ions in the CaM N-terminal, we considered an increase in the rate constant for the dissociation of the complex PDE/Ca^{2+}/CaM of 100-fold ($k_b = 1$ s^{-1})[61]. The full description of the reactions and parameters are listed in Supplementary Table I (React80-Reac85).

PDE, as well as other CaM targets, modulates the affinity of CaM for Ca^{2+}. To incorporate this effect, we recalculated the rate constants for Ca^{2+} dissociation from CaM bound to PDE1 considering an overall increase in the affinity of Ca^{2+} for CaM in the presence of PDE of 25-fold [62]. No alteration was assumed for the rate constants of association between Ca^{2+} and CaM bound to PDE1. Therefore, considering equations (5)-(8) and the values of k_I/k_{II} (0.015 $\mu mol^{-1}.L$), k_{III}/k_{IV} (0.04 $\mu mol^{-1}.L$) for the interaction of Ca^{2+} to CaM in absence of PDE1[32], the values of the microscopic constants of affinity for the binding of Ca^{2+} to CaM in presence of PDE1 were calculate as k_I/k_{II} (0.3750 $\mu mol^{-1}.L$), k_{III}/k_{IV} (1 $\mu mol^{-1}.L$). The rate

constants for Ca^{2+} association with CaM used in the model were k_{f_I} and k_{f_II} = 750 µmol $^{-1}$.L.s $^{-1}$, k_{f_III} = 800 µmol $^{-1}$.L.s $^{-1}$, and k_{f_IV} = 204 µmol $^{-1}$.L.s $^{-1}$ [32] (the terms I, II, III and IV indicate the Ca^{2+}-binding sites I, II, III and IV, respectively). With these constants of affinity and the rate constants for the association of Ca^{2+} to the complex, the rate constants for the dissociation of Ca^{2+}, in absence of cooperativity (only one ion bound to a given domain), from CaM associated to PDE1 were calculated as k_{b_I} and k_{b_II} = 2000 s^{-1}, k_{b_III} = 800 s^{-1}, and k_{b_IV} = 204 s^{-1}. The rate constants for the unbinding of the first ion when two ions are bound to a pair of sites located on given domain of CaM associated to PDE1 were calculated considering the coupling constants k_n (80 µmol $^{-1}$.L), and k_c (200 µmol $^{-1}$.L): k_{b_I} and k_{b_II} = 25 s^{-1}, k_{b_III} = 4 s^{-1}, and k_{b_IV} = 1.02 s^{-1} (Supplementary Figure S11C, Supplementary Table I: Reac86-Reac92).

cAMP dynamics and PKA

The balance between PDE1 and AC activity is fundamental to control the intracellular level of cAMP, one of the most important intracellular second messengers. The main target of cAMP is the cAMP-dependent protein kinase (PKA) [63,64], an enzyme that frequently opposes CaN action. AC produces cAMP with a relative high basal rate[65] that is further accelerated by the rise of Ca^{2+} and the formation of Ca^{2+}/CaM [51,55,66,67]. To implement this process, we used the same set of reactions to simulated the production of cAMP by AC in the presence or absence Ca^{2+}/CaM, but assumed that the rate constants of these reactions are speeded by Ca^{2+}/CaM [51,55,66,67].

In contrast to AC, PDE1 had a low basal activity in our model. The degradation of cAMP catalysed by PDE1 required the presence of Ca^{2+}/CaM [59,68]. In consequence, in addition to the action of PDE1, we used a constant degradation reaction of cAMP to

counteract the basal activity of AC and sustains the basal concentration of cAMP ([cAMP]) around 100 $nmol.L^{-1}$ [69]. In neurons, elevations of [cAMP] can reach up to 3 $\mu mol.L^{-1}$ [69], but require the simultaneous stimulation of AC by Ca^{2+}/CaM and protein $G_{\alpha s}$[66], which was not implemented in the model. Thus, the rise of Ca^{2+} in the model promoted peak elevations of [cAMP] around 500-600 $nmol.L^{-1}$. The complete description of the reactions and parameters used in the production and degradation of cAMP is listed in Supplementary Table I (Reac93-Reac97).

The rise of [cAMP] in the model regulated the activity of PKA. At rest, PKA is a tetrameric enzyme with two catalytic (C) subunits and a regulatory (R) subunit dimer (R_2) [64,70]. This holoenzyme complex kept PKA C subunits in an inactive state in absence of cAMP, which binds to cyclic nucleotide-binding domains (CNB) located in PKA R subunits [64,70]. Structurally, each R subunit contains two tandem CNB domains (CNB-A and CNB-B) [64,70]. The binding of cAMP to the two CNBs in each R subunit promotes a conformational change that leads to the release of the two active C subunits from PKA holoenzyme [64,70]. The R subunits occur in two distinct classes (RI, RII), each having an α and β isoforms [6,7]. RIIβ is the predominant isoform in the brain and the tetramer $(RII\beta)_2C_2$ has the highest cAMP requirement in comparison to other PKA holoenzymes [6,7].

The crystalized structure of the full-length $(RII\beta)_2C_2$ solved recently indicates that each RIIβ subunit of the tetrameric PKA contacts the neighbouring R:C complex[6]. The physical contacts among different subunits in the tetrameric holoenzyme are responsible for PKA allosteric activation [6]. Several groups have developed computational models to simulate PKA activity[71,72], but most of these models simulated PKA as a dimer of heterodimers, which is not supported by the experimental findings that clearly demonstrate that there is communication between the subunits of the tetrameric PKA during its activation [6,73]. To

simulate PKA, we assumed that it is structurally composed by a dimer of two R subunits $(RII\beta)_2$ coupled to two C subunits. The binding/unbinding of each C subunit to a RIIβ subunit of the dimer $(RII\beta)_2$ was implemented as independent events in the absence of cAMP.

Endogenous PKA is autophosphorylated in the cells, which affects the affinities between the C and RIIβ subunits[10]. The phosphorylation of the RIIβ subunits by the C subunits was modelled as a first order reaction. PKA is largely in its autophosphorylated state in our model. To simulate the binding/unbinding of each C subunit to the phosphorylated RIIβ subunits, we used the same reactions implemented for the non-phosphorylated PKA, but different rate constants[10].

cAMP can access both the CNB-A and CNB-B of the RIIβ subunits in the apoenzyme[7]. In consequence, we simulated the interaction of cAMP to each cAMP-binding site as random and independent events. The affinities and the rate constants observed for the binding/unbinding of cAMP to the isolated $(RII\beta)_2$ dimer are different from the parameters reported for the full holoenzyme [74–76]. We assumed that each CNB of the tetrameric PKA has a 3-fold lower affinity for cAMP than the CNBs from the isolated $(RII\beta)_2$ dimer [7,76]. The phosphorylation of the RIIβ subunits had no effects on their affinities for cAMP.

The binding of two cAMP molecules to each R subunit of the inactive PKA induces a decrease in the affinity between the C and RIIβ subunits, which leads to the dissociation of the holoenzyme into two free C subunits and the $(RII\beta)_2$ dimer [64,70]. The K_D for the interaction of the C and RIIβ subunit is 0.14 $nmol.L^{-1}$ in absence of cAMP, and approximately 15-fold greater in presence of four molecules of cAMP [7], which we implemented by changing the rate constant for the complex $(RII\beta)_2C_2$ dissociation. The activation of PKA fully saturated with cAMP is highly cooperative (n_{Hill} = 1.7). To capture this property, we assumed that, after the release of the first C subunit from the holoenzyme $(RII\beta)_2C_2$, the second C subunit

was released with a faster rate constant. This cooperativity was implemented only for PKA fully saturated with cAMP.

Mutations of the CNB-B domain promote a decrease in the $K_{1/2}$ for PKA activation, which is due mainly to a change in the rate constant (k_b) for the dissociation of the complex $(RII\beta)_2C_2$ [6,7]. We used this information to define the rate constants for the release of the C subunits from the tetrameric PKA in the absence of cAMP bound to the CNB-B sites: we kept k_f for the complex association unchanged and multiplied the k_b by twenty [6,7]. In contrast, mutations in the CNB-A domains promotes a strong increase in the $K_{1/2}$ for PKA activation[7], which we took into account to implement the dissociation/association between the C and the R subunits in absence of cAMP bound to the CNB-A sites. The rate constant for this dissociation was defined as the k_b for the release of the C subunits from the tetramer $(RII\beta)_2C_2$ divided by fifth [7], k_f was kept unchanged. We simulated the interactions between the C and the phosphorylated RIIβ subunits using the same chemical reactions implemented for the non-phosphorylated RIIβ subunits, but different rate constants[10].

To validate the thermodynamics properties of the PKA model implemented, we fitted its dose-response curves (equation (1) of the main text) of activation as a function of [cAMP]. Contrarily to the classical view of PKA activation, the release of the C subunits from the $(RII\beta)_2$ dimer is only partial even in the presence of high excess of [cAMP][77,78]. The presence of excess of cAMP in combination with excess of a PKA substrate increases the dissociation of the holoenzyme, but, even under these conditions, the maximum dissociation observed is around 80% of the total amount of PKA[77]. In our model, in absence of substrates, the maximum activation of PKA was around 0.3 (maximum activity was normalized to 1) with a n_{Hill} of 1.46 and $K_{1/2}$ of 0.7 $\mu mol.L^{-1}$ (Supplementary Figure S9A). We did not find any experimental curve of PKA activation as a function of cAMP in the absence of substrates to

compare with our results. In the presence of the substrate Kemptide (concentration of Kemptide = 1000 μmol.L^{-1} and 1 μmol.L^{-1} of PKA), the maximum PKA activity was approximately 0.8 as observed experimentally [77]. The n_{hill} obtained was 1.55 and the $K_{1/2}$ was 0.57 μmol.L^{-1} (Supplementary Figure S9A). Experimental curves obtained using different concentrations of Kemptide reported n_{hill} of 1.7 and $K_{1/2}$ around 0.58-0.6 μmol.L^{-1} [6,7].

To validate the kinetics of the PKA model, we verified the time course for the association of the holoenzyme in absence of cAMP and the time course for its dissociation after the addition of cAMP (500 μmol.L^{-1} of cAMP and 1 μmol.L^{-1} of PKA) and compared the results with experimental data. For this analysis, we started the simulations with the C subunits fully dissociated from the $(RII\beta)_2$ dimers and verified the time course for the formation of the holoenzyme. After 400 s of simulated time, we modelled a release of cAMP to verify the time course of the holoenzyme dissociation. Initially, our model presented a slower time course for the dissociation of the C subunits from PKA in comparison to experimental data. To overcome this problem, we multiplied all the rate constants of dissociation/association of the C subunits from the $(RII\beta)_2$ dimers bound to cAMP by two, which resulted in a time course of PKA dissociation very similar to a curve extracted from the experimental literature[8] (Supplementary Figure S9B). This symmetrical alteration promoted no change in the thermodynamics properties of the model. The full description of the reactions and parameters used to simulate the association and dissociation of PKA subunits are listed in the Supplementary Table I (Reac98-Reac120).

Additional model details

To verify the putative consequences of the different patterns of AC, PDE and, CaN to signalling pathways, we implemented a hypothetical target (termed subs) that act as a

substrate of PKA and, in its phosphorylated state, of CaN. The reactions of phosphorylation and dephosphorylation of the subs by PKA and CaN, respectively, were implemented using exactly the same rate constants, which were based on the rate constants for the phosphorylation of Kemptide by PKA (Supplementary Table I, Reac121).

The simulations began with the the total amount of subs completely phosphorylated. Then, we stimulated the model using 100 glutamate pulses released at 1 Hz and 10 Hz to verified how the combined activity of CaN, AC, PDE1 and PKA regulates the dephosphorylation of subs (Fig. 5G-H).

Data Analysis

We calculated the integral of each single pulse of calcium by the summation of [Ca^{2+}] amplitudes during the occurrence of the pulse multiplied by dt that is the time step of the results (1 ms). Then, we plotted the cumulative sum of the integrated calcium pulses (Cum.∫[Ca^{2+}].dt) versus the activity of each enzyme for each value of V_m and frequency of stimulation.

All fittings used a confidence interval of 95% and were calculated with the Matlab toolbox cftool. The decay time constants were obtained by fitting the simulated results with a monoexponential or a biexponential function.

The dose-response curves were fitted with the Hill equation described in the main text (equation 1). The concentrations of CaM used during the dose-response curves were in excess and, consequently, should not influence $K_{1/2}$ and n_{Hill}. Thus, in this situations, Ca^{2+} was the only limiting factor. Hippocampal neurons have very high CaM content (>20 µM[79,80]). There is a range of estimated values between 20 µM to 100 µM in the literature. The exact concentration of CaM in dendritic spines is unknown.

References

1. Feng, B. & Stemmer, P. M. Ca2+ binding site 2 in calcineurin-B modulates calmodulin-dependent calcineurin phosphatase activity. *Biochemistry (Mosc.)* **40,** 8808–14 (2001).
2. Wittenberg, G. M. & Wang, S. S.-H. Malleability of spike-timing-dependent plasticity at the CA3-CA1 synapse. *J. Neurosci. Off. J. Soc. Neurosci.* **26,** 6610–6617 (2006).
3. Nevian, T. & Sakmann, B. Spine Ca2+ signaling in spike-timing-dependent plasticity. *J Neurosci* **26,** 11001–13 (2006).
4. Bi, G. & Poo, M. Synaptic modification by correlated activity: Hebb's postulate revisited. *Annu Rev Neurosci* **24,** 139–66 (2001).
5. Gallagher, S. C. *et al.* There is communication between all four Ca(2+)-bindings sites of calcineurin B. *Biochemistry (Mosc.)* **40,** 12094–102 (2001).
6. Zhang, P. *et al.* Structure and allostery of the PKA RIIβ tetrameric holoenzyme. *Science* **335,** 712–6 (2012).
7. Zawadzki, K. M. & Taylor, S. S. cAMP-dependent protein kinase regulatory subunit type IIbeta: active site mutations define an isoform-specific network for allosteric signaling by cAMP. *J Biol Chem* **279,** 7029–36 (2004).
8. Zhang, P. *et al.* Single Turnover Autophosphorylation Cycle of the PKA RIIβ Holoenzyme. *PLOS Biol.* **13,** e1002192 (2015).
9. Clarke, R. J. & Johnson, J. W. Voltage-dependent gating of NR1/2B NMDA receptors. *J Physiol* **586,** 5727–41 (2008).
10. Clarke, R. J., Glasgow, N. G. & Johnson, J. W. Mechanistic and structural determinants of NMDA receptor voltage-dependent gating and slow Mg2+ unblock. *J Neurosci* **33,** 4140–50 (2013).

11. Erreger, K., Dravid, S. M., Banke, T. G., Wyllie, D. J. & Traynelis, S. F. Subunit-specific gating controls rat NR1/NR2A and NR1/NR2B NMDA channel kinetics and synaptic signalling profiles. *J Physiol* **563,** 345–58 (2005).

12. Paoletti, P., Bellone, C. & Zhou, Q. NMDA receptor subunit diversity: impact on receptor properties, synaptic plasticity and disease. *Nat Rev Neurosci* **14,** 383–400 (2013).

13. Lüscher, C. & Malenka, R. C. NMDA receptor-dependent long-term potentiation and long-term depression (LTP/LTD). *Cold Spring Harb Perspect Biol* **4,** (2012).

14. Henneberger, C., Bard, L., King, C., Jennings, A. & Rusakov, D. A. NMDA receptor activation: two targets for two co-agonists. *Neurochem Res* **38,** 1156–62 (2013).

15. Gray, J. A. *et al.* Distinct modes of AMPA receptor suppression at developing synapses by GluN2A and GluN2B: single-cell NMDA receptor subunit deletion in vivo. *Neuron* **71,** 1085–101 (2011).

16. Bellone, C. & Nicoll, R. A. Rapid bidirectional switching of synaptic NMDA receptors. *Neuron* **55,** 779–85 (2007).

17. Chen, X. *et al.* Organization of the core structure of the postsynaptic density. *Proc Natl Acad Sci U A* **105,** 4453–8 (2008).

18. Ascher, P. & Nowak, L. The role of divalent cations in the N-methyl-D-aspartate responses of mouse central neurones in culture. *J Physiol* **399,** 247–66 (1988).

19. Banke, T. G. & Traynelis, S. F. Activation of NR1/NR2B NMDA receptors. *Nat Neurosci* **6,** 144–52 (2003).

20. Sabatini, B. L., Oertner, T. G. & Svoboda, K. The life cycle of Ca(2+) ions in dendritic spines. *Neuron* **33,** 439–52 (2002).

21. Schorge, S., Elenes, S. & Colquhoun, D. Maximum likelihood fitting of single channel

NMDA activity with a mechanism composed of independent dimers of subunits. *J Physiol* **569,** 395–418 (2005).

22. Jahr, C. E. & Stevens, C. F. Calcium permeability of the N-methyl-D-aspartate receptor channel in hippocampal neurons in culture. *Proc Natl Acad Sci U A* **90,** 11573–7 (1993).
23. Garaschuk, O., Schneggenburger, R., Schirra, C., Tempia, F. & Konnerth, A. Fractional Ca2+ currents through somatic and dendritic glutamate receptor channels of rat hippocampal CA1 pyramidal neurones. *J Physiol* **491 (Pt 3),** 757–72 (1996).
24. Kovalchuk, Y., Eilers, J., Lisman, J. & Konnerth, A. NMDA receptor-mediated subthreshold Ca(2+) signals in spines of hippocampal neurons. *J Neurosci* **20,** 1791–9 (2000).
25. Schneggenburger, R. Simultaneous measurement of Ca2+ influx and reversal potentials in recombinant N-methyl-D-aspartate receptor channels. *Biophys J* **70,** 2165–74 (1996).
26. Faeder, J. R., Blinov, M. L. & Hlavacek, W. S. Rule-based modeling of biochemical systems with BioNetGen. *Methods Mol Biol* **500,** 113–67 (2009).
27. Chin, D. & Means, A. R. Calmodulin: a prototypical calcium sensor. *Trends Cell Biol* **10,** 322–8 (2000).
28. Xia, Z. & Storm, D. R. The role of calmodulin as a signal integrator for synaptic plasticity. *Nat Rev Neurosci* **6,** 267–76 (2005).
29. Linse, S., Helmersson, A. & Forsén, S. Calcium binding to calmodulin and its globular domains. *J Biol Chem* **266,** 8050–4 (1991).
30. Shea, M. A., Verhoeven, A. S. & Pedigo, S. Calcium-induced interactions of calmodulin domains revealed by quantitative thrombin footprinting of Arg37 and Arg106. *Biochemistry (Mosc.)* **35,** 2943–57 (1996).
31. Boschek, C. B., Squier, T. C. & Bigelow, D. J. Disruption of interdomain interactions

via partial calcium occupancy of calmodulin. *Biochemistry (Mosc.)* **46,** 4580–8 (2007).

32. Antunes, G., Sebastião, A. M. & Simoes de Souza, F. M. Mechanisms of Regulation of Olfactory Transduction and Adaptation in the Olfactory Cilium. *PLoS One* **9,** e105531 (2014).
33. Brini, M. & Carafoli, E. Calcium pumps in health and disease. *Physiol Rev* **89,** 1341–78 (2009).
34. Caride, A. J., Filoteo, A. G., Penniston, J. T. & Strehler, E. E. The plasma membrane Ca2+ pump isoform 4a differs from isoform 4b in the mechanism of calmodulin binding and activation kinetics: implications for Ca2+ signaling. *J Biol Chem* **282,** 25640–8 (2007).
35. Osborn, K. D., Zaidi, A., Mandal, A., Urbauer, R. J. & Johnson, C. K. Single-molecule dynamics of the calcium-dependent activation of plasma-membrane Ca2+-ATPase by calmodulin. *Biophys J* **87,** 1892–9 (2004).
36. Penheiter, A. R., Filoteo, A. G., Penniston, J. T. & Caride, A. J. Kinetic analysis of the calmodulin-binding region of the plasma membrane calcium pump isoform 4b. *Biochemistry (Mosc.)* **44,** 2009–20 (2005).
37. Slaughter, B. D., Urbauer, R. J., Urbauer, J. L. & Johnson, C. K. Mechanism of calmodulin recognition of the binding domain of isoform 1b of the plasma membrane Ca(2+)-ATPase: kinetic pathway and effects of methionine oxidation. *Biochemistry (Mosc.)* **46,** 4045–54 (2007).
38. Sun, H. & Squier, T. C. Ordered and cooperative binding of opposing globular domains of calmodulin to the plasma membrane Ca-ATPase. *J Biol Chem* **275,** 1731–8 (2000).
39. Liyanage, M. R., Zaidi, A. & Johnson, C. K. Fluorescence polarization assay for calmodulin binding to plasma membrane Ca2+-ATPase: dependence on enzyme and

Ca2+ concentrations. *Anal Biochem* **385,** 1–6 (2009).

40. Stemmer, P. M. & Klee, C. B. Dual calcium ion regulation of calcineurin by calmodulin and calcineurin B. *Biochemistry (Mosc.)* **33,** 6859–66 (1994).

41. Feng, B. & Stemmer, P. M. Interactions of calcineurin A, calcineurin B, and Ca2+. *Biochemistry (Mosc.)* **38,** 12481–9 (1999).

42. Yang, S. A. & Klee, C. B. Low affinity Ca2+-binding sites of calcineurin B mediate conformational changes in calcineurin A. *Biochemistry (Mosc.)* **39,** 16147–54 (2000).

43. Shen, X. *et al.* The secondary structure of calcineurin regulatory region and conformational change induced by calcium/calmodulin binding. *J Biol Chem* **283,** 11407–13 (2008).

44. Klee, C. B., Ren, H. & Wang, X. Regulation of the calmodulin-stimulated protein phosphatase, calcineurin. *J Biol Chem* **273,** 13367–70 (1998).

45. Quintana, A. R., Wang, D., Forbes, J. E. & Waxham, M. N. Kinetics of calmodulin binding to calcineurin. *Biochem Biophys Res Commun* **334,** 674–80 (2005).

46. O'Donnell, S. E., Yu, L., Fowler, C. A. & Shea, M. A. Recognition of β-calcineurin by the domains of calmodulin: thermodynamic and structural evidence for distinct roles. *Proteins* **79,** 765–86 (2011).

47. Bayley, P. M., Findlay, W. A. & Martin, S. R. Target recognition by calmodulin: dissecting the kinetics and affinity of interaction using short peptide sequences. *Protein Sci* **5,** 1215–28 (1996).

48. Brown, S. E., Martin, S. R. & Bayley, P. M. Kinetic control of the dissociation pathway of calmodulin-peptide complexes. *J Biol Chem* **272,** 3389–97 (1997).

49. Peersen, O. B., Madsen, T. S. & Falke, J. J. Intermolecular tuning of calmodulin by target peptides and proteins: differential effects on Ca2+ binding and implications for kinase

activation. *Protein Sci* **6,** 794–807 (1997).

50. Halls, M. L. & Cooper, D. M. Regulation by Ca2+-signaling pathways of adenylyl cyclases. *Cold Spring Harb Perspect Biol* **3,** a004143 (2011).
51. Gao, Z. H. *et al.* Activation of four enzymes by two series of calmodulin mutants with point mutations in individual Ca2+ binding sites. *J Biol Chem* **268,** 20096–104 (1993).
52. Masada, N., Schaks, S., Jackson, S. E., Sinz, A. & Cooper, D. M. Distinct mechanisms of calmodulin binding and regulation of adenylyl cyclases 1 and 8. *Biochemistry (Mosc.)* **51,** 7917–29 (2012).
53. Iqbal, Z. & Sze, P. Y. [125I]calmodulin binding to synaptic plasma membrane from rat brain: kinetic and Arrhenius analysis. *Neurochem Res* **18,** 897–905 (1993).
54. Cooper, D. M. Regulation and organization of adenylyl cyclases and cAMP. *Biochem J* **375,** 517–29 (2003).
55. Guillou, J. L., Nakata, H. & Cooper, D. M. Inhibition by calcium of mammalian adenylyl cyclases. *J Biol Chem* **274,** 35539–45 (1999).
56. Conti, M. & Beavo, J. Biochemistry and physiology of cyclic nucleotide phosphodiesterases: essential components in cyclic nucleotide signaling. *Annu Rev Biochem* **76,** 481–511 (2007).
57. Goraya, T. A. & Cooper, D. M. Ca2+-calmodulin-dependent phosphodiesterase (PDE1): current perspectives. *Cell Signal* **17,** 789–97 (2005).
58. Cox, J. A., Malnoë, A. & Stein, E. A. Regulation of brain cyclic nucleotide phosphodiesterase by calmodulin. A quantitative analysis. *J Biol Chem* **256,** 3218–22 (1981).
59. Huang, C. Y., Chau, V., Chock, P. B., Wang, J. H. & Sharma, R. K. Mechanism of activation of cyclic nucleotide phosphodiesterase: requirement of the binding of four

Ca2+ to calmodulin for activation. *Proc Natl Acad Sci U A* **78,** 871–4 (1981).

60. Goraya, T. A. *et al.* Kinetic properties of Ca2+/calmodulin-dependent phosphodiesterase isoforms dictate intracellular cAMP dynamics in response to elevation of cytosolic Ca2+. *Cell Signal* **20,** 359–74 (2008).

61. Zhang, M., Li, M., Wang, J. H. & Vogel, H. J. The effect of Met-->Leu mutations on calmodulin's ability to activate cyclic nucleotide phosphodiesterase. *J Biol Chem* **269,** 15546–52 (1994).

62. Olwin, B. B. & Storm, D. R. Calcium binding to complexes of calmodulin and calmodulin binding proteins. *Biochemistry (Mosc.)* **24,** 8081–6 (1985).

63. Cheng, X., Ji, Z., Tsalkova, T. & Mei, F. Epac and PKA: a tale of two intracellular cAMP receptors. *Acta Biochim Biophys Sin Shanghai* **40,** 651–62 (2008).

64. Taylor, S. S., Zhang, P., Steichen, J. M., Keshwani, M. M. & Kornev, A. P. PKA: lessons learned after twenty years. *Biochim Biophys Acta* **1834,** 1271–8 (2013).

65. Ferguson, G. D. & Storm, D. R. Why calcium-stimulated adenylyl cyclases? *Physiol. Bethesda* **19,** 271–6 (2004).

66. Wayman, G. A. *et al.* Synergistic activation of the type I adenylyl cyclase by Ca2+ and Gs-coupled receptors in vivo. *J. Biol. Chem.* **269,** 25400–25405 (1994).

67. Masada, N., Ciruela, A., Macdougall, D. A. & Cooper, D. M. Distinct mechanisms of regulation by Ca2+/calmodulin of type 1 and 8 adenylyl cyclases support their different physiological roles. *J Biol Chem* **284,** 4451–63 (2009).

68. Sharma, R. K. & Kalra, J. Characterization of calmodulin-dependent cyclic nucleotide phosphodiesterase isoenzymes. *Biochem J* **299 (Pt 1),** 97–100 (1994).

69. Mironov, S. L. *et al.* Imaging cytoplasmic cAMP in mouse brainstem neurons. *BMC Neurosci* **10,** 29 (2009).

70. Taylor, S. S., Ilouz, R., Zhang, P. & Kornev, A. P. Assembly of allosteric macromolecular switches: lessons from PKA. *Nat Rev Mol Cell Biol* **13,** 646–58 (2012).

71. Bhalla, U. S. & Iyengar, R. Emergent properties of networks of biological signaling pathways. *Science* **283,** 381–7 (1999).

72. Kotaleski, J. H. & Blackwell, K. T. Modelling the molecular mechanisms of synaptic plasticity using systems biology approaches. *Nat Rev Neurosci* **11,** 239–51 (2010).

73. Byeon, I. J. *et al.* Allosteric communication between cAMP binding sites in the RI subunit of protein kinase A revealed by NMR. *J Biol Chem* **285,** 14062–70 (2010).

74. Ogreid, D. & Døskeland, S. O. The kinetics of association of cyclic AMP to the two types of binding sites associated with protein kinase II from bovine myocardium. *FEBS Lett* **129,** 287–92 (1981).

75. Ogreid, D. & Døskeland, S. O. The kinetics of the interaction between cyclic AMP and the regulatory moiety of protein kinase II. Evidence for interaction between the binding sites for cyclic AMP. *FEBS Lett* **129,** 282–6 (1981).

76. Dao, K. K. *et al.* Epac1 and cAMP-dependent protein kinase holoenzyme have similar cAMP affinity, but their cAMP domains have distinct structural features and cyclic nucleotide recognition. *J Biol Chem* **281,** 21500–11 (2006).

77. Trewhella, J. Protein kinase A targeting and activation as seen by small-angle solution scattering. *Eur J Cell Biol* **85,** 655–62 (2006).

78. Vigil, D., Blumenthal, D. K., Brown, S., Taylor, S. S. & Trewhella, J. Differential effects of substrate on type I and type II PKA holoenzyme dissociation. *Biochemistry (Mosc.)* **43,** 5629–36 (2004).

79. Biber, A., Schmid, G. & Hempel, K. Calmodulin content in specific brain areas. *Exp Brain Res* **56,** 323–6 (1984).

80. Kakiuchi, S. *et al.* Quantitative determinations of calmodulin in the supernatant and particulate fractions of mammalian tissues. *J Biochem* **92,** 1041–8 (1982).

81. Clements, J. D., Lester, R. A., Tong, G., Jahr, C. E. & Westbrook, G. L. The time course of glutamate in the synaptic cleft. *Science* **258,** 1498–501 (1992).

82. Moore, M. J., Adams, J. A. & Taylor, S. S. Structural basis for peptide binding in protein kinase A. Role of glutamic acid 203 and tyrosine 204 in the peptide-positioning loop. *J. Biol. Chem.* **278,** 10613–10618 (2003).

83. Lew, J., Taylor, S. S. & Adams, J. A. Identification of a partially rate-determining step in the catalytic mechanism of cAMP-dependent protein kinase: a transient kinetic study using stopped-flow fluorescence spectroscopy. *Biochemistry (Mosc.)* **36,** 6717–6724 (1997).

84. Sims, P. C. *et al.* Electronic Measurements of Single-Molecule Catalysis by cAMP-Dependent Protein Kinase A. *J. Am. Chem. Soc.* **135,** 7861–7868 (2013).

Supplementary Table I: Parameters of the model

Reactions ID	Species/Reactions	Parameters	Notes	References
	Volume of the compartment	0.18e-15 L		
	Number of NMDAR	20 (13 GluN1/GluN2A and 7 GluN1/GluN2B)		[15–17]
	Number of PMCA	200	This parameter was defined to reproduce the decay time constant of synaptically evoked Ca^{2+} transients	This paper
	Number of NCX	200	This parameter	This paper

			was defined to reproduce the decay time constant of synaptically evoked Ca^{2+} transients	
	Number of channels of leak	12	Parameter used to sustain the basal $[Ca^{2+}]$.	This paper
	Concentration of CaM	40 μmol.L^{-1}		[79,80]
	Concentration of UB	40 μmol.L^{-1}	Defined to reproduce a buffer capacity	This paper

			of 20	
	Concentration of PDE/CaN/AC	1 μmol.L^{-1}	Equimolar concentration used for comparative analysis	This paper
	Basal concentration of cAMP	~ 100 nmol.L^{-1}		[69]
	Concentration of PKA	1 μmol.L^{-1}		This paper
	Concentration of PKA/CaN substrate (subs)	100 μmol.L^{-1}		This paper
Reac1	$Glu + NMDAR \underset{k_b}{\overset{2k_f}{\rightleftharpoons}} Glu.NMDAR$	$k_{f1(2A)}$ = 63.2 μmol^{-1}.L.s^{-1}<br>$k_{b1(2A)}$ = 1010 s^{-1}<br>$k_{f1(2B)}$ = 5.66 μmol^{-1}.L.s^{-1}<br>$k_{b1(2B)}$ = 28.1s^{-1}	Interaction between glutamate (Glu) and NMDARs, the rate constants are	[9–11]

			given for the two types of NMDARs included in the model. The terms (2A) and (2B) refer to GluN1/GluN2A and GluN1/GluN2B NMDARs, respectively.	
Reac2	$Glu + Glu.NMDAR \underset{2k_b}{\overset{k_f}{\rightleftharpoons}} Glu_2.NMDAR$	$k_{f1(2A)}$ = 63.2 µmol^{-1}.L.s^{-1}<br>$k_{b1(2A)}$ = 1010 s^{-1}		[9–11]

		k_{f1_2B} = 5.66 µmol^{-1}.L.s^{-1}<br>k_{b1_2B} = 28.1s^{-1}		
Reac3	$Glu_2.NMDAR \underset{k_b}{\overset{k_f}{\rightleftharpoons}} Glu_2.NMDAR_{d1}$	$k_{f2(2A)}$ = 107 s^{-1}<br>$k_{b2(2A)}$ = 3.5 s^{-1}<br>$k_{f2(2B)}$ = 550 s^{-1}<br>$k_{b2(2B)}$ = 81.4 s^{-1}	The term d1 indicates the desensitized receptor.	[9–11]
Reac4	$Glu_2.NMDAR \underset{k_b}{\overset{k_f}{\rightleftharpoons}} Glu_2.NMDAR_{d2}$	$k_{f3(2A)}$ = 46.7 s^{-1}<br>$k_{b3(2A)}$ = 0.41 s^{-1}<br>$k_{f3(2B)}$ = 112 s^{-1}<br>$k_{b3(2B)}$ = 0.91 s^{-1}	The term d2 indicates the desensitized receptor.	[9–11]
Reac5	$Glu_2.NMDAR \underset{k_b}{\overset{k_f}{\rightleftharpoons}} Glu_2.NMDAR_{f}$	$k_{f4(2A)}$ = 3140 s^{-1}<br>$k_{b4(2A)}$ = 174 s^{-1}<br>$k_{f4(2B)}$ = 2836 s^{-1}<br>$k_{b4(2B)}$ = 175 s^{-1}		[9–11]

Reac6	$Glu_2.NMDAR \underset{k_b}{\overset{k_f}{\rightleftharpoons}} Glu_2.NMDAR_s$	$k_{f5(2A)} = 230\exp\left(\frac{V_m-100}{175}\right)s^{-1}$<br>$k_{b5(2A)}$ = 178 s^{-1}<br>$k_{f5(2B)} = 48\exp\left(\frac{V_m-100}{175}\right)s^{-1}$<br>$k_{b5(2B)}$ = 230 s^{-1}		9,10
Reac7	$Glu_2.NMDAR_f \underset{k_b}{\overset{k_f}{\rightleftharpoons}} Glu_2.NMDAR_o$	$k_{f5(2A)} = 230\exp\left(\frac{V_m-100}{175}\right)s^{-1}$<br>$k_{b5(2A)}$ = 178 s^{-1}<br>$k_{f5(2B)} = 48\exp\left(\frac{V_m-100}{175}\right)s^{-1}$<br>$k_{b5(2B)}$ = 230 s^{-1}	The term O indicates the open receptor.	9,10
Reac8	$Glu_2.NMDAR_s \underset{k_b}{\overset{k_f}{\rightleftharpoons}} Glu_2.NMDAR_o$	$k_{f4(2A)}$ = 3140 s^{-1}<br>$k_{b4(2A)}$ = 174 s^{-1}<br>$k_{f4(2B)}$ = 2836 s^{-1}<br>$k_{b4(2B)}$ = 175 s^{-1}		9–11
Reac9	$Glu_2.NMDAR_o \underset{k_{b(V_m)}}{\overset{k_{f(V_m)}}{\rightleftharpoons}} Glu_2.NMDAR_o^{Mg}$	$k_{f(V_m)} = 610\exp\left(\frac{-V_m}{17}\right)[Mg^{2+}]s^{-1}$<br>$[Mg^{2+}]$ = 1 mmol.L^{-1}		18

		$k_{b(V_m)} = 5400\exp\left(\frac{V_m}{47}\right)s^{-1}$		
Reac10	$Glu + NMDAR^{Mg} \underset{k_b}{\overset{2k_f}{\rightleftharpoons}} Glu.NMDAR^{Mg}$	$k_{f1(2A)}$ = 63.2 µmol^{-1}.L.s^{-1}<br>$k_{b1(2A)}$ = 1010 s^{-1}<br>$k_{f1(2B)}$ = 5.66 µmol^{-1}.L.s^{-1}<br>$k_{b1(2B)}$ = 38.1s^{-1}	NMDAR blocked by Mg^{2+} ($NMDAR^{Mg}$)	9–11
Reac11	$Glu + Glu.NMDAR^{Mg} \underset{2k_b}{\overset{k_f}{\rightleftharpoons}} Glu_2.NMDAR^{Mg}$	$k_{f1(2A)}$ = 63.2 µmol^{-1}.L.s^{-1}<br>$k_{b1(2A)}$ = 1010 s^{-1}<br>$k_{f1(2B)}$ = 5.66 µmol^{-1}.L.s^{-1}<br>$k_{b1(2B)}$ = 38.1s^{-1}		9–11
Reac12	$Glu_2.NMDAR^{Mg} \underset{k_b}{\overset{k_f}{\rightleftharpoons}} Glu_2.NMDAR^{Mg}_{d1}$	$k_{f2(2A)}$ = 107 s^{-1}<br>$k_{b2(2A)}$ = 3.5 s^{-1}<br>$k_{f2(2B)}$ = 550 s^{-1}<br>$k_{b2(2B)}$ = 81.4 s^{-1}		9–11
Reac13	$Glu_2.NMDAR \underset{k_b}{\overset{k_f}{\rightleftharpoons}} Glu_2.NMDAR^{Mg}_{d2}$	$k_{f3(2A)}$ = 46.7 s^{-1}		9–11

		$k_{b3(2A)} = 0.41\ s^{-1}$<br>$k_{f3(2B)} = 112\ s^{-1}$<br>$k_{b3(2B)} = 0.91\ s^{-1}$		
Reac14	$Glu_2.NMDAR \underset{k_b}{\overset{k_f}{\rightleftharpoons}} Glu_2.NMDAR_f^{Mg}$	$k_{f4(2A)} = 3140\ s^{-1}$<br>$k_{b4(2A)} = 174\ s^{-1}$<br>$k_{f4(2B)} = 2836\ s^{-1}$<br>$k_{b4(2B)} = 175\ s^{-1}$		9–11
Reac15	$Glu_2.NMDAR \underset{k_b}{\overset{k_f}{\rightleftharpoons}} Glu_2.NMDAR_s^{Mg}$	$k_{f5(2A)} = 230\exp\left(\frac{V_m - 100}{175}\right)s^{-1}$<br>$k_{b5(2A)} = 178\ s^{-1}$<br>$k_{f5(2B)} = 48\exp\left(\frac{V_m - 100}{175}\right)s^{-1}$<br>$k_{b5(2B)} = 230\ s^{-1}$		9,10
Reac16	$Glu_2.NMDAR_f^{Mg} \underset{k_b}{\overset{k_f}{\rightleftharpoons}} Glu_2.NMDAR_o^{Mg}$	$k_{f5(2A)} = 230\exp\left(\frac{V_m - 100}{175}\right)s^{-1}$<br>$k_{b5(2A)} = 178\ s^{-1}$<br>$k_{f5(2B)} = 48\exp\left(\frac{V_m - 100}{175}\right)s^{-1}$		9,10

		$k_{b5(2B)}$ = 230 s^{-1}		
Reac17	$Glu_2.NMDAR_s^{Mg} \underset{k_b}{\overset{k_f}{\rightleftharpoons}} Glu_2.NMDAR_o^{Mg}$	$k_{f4(2A)}$ = 3140 s^{-1}<br>$k_{b4(2A)}$ = 174 s^{-1}<br>$k_{f4(2B)}$ = 2836 s^{-1}<br>$k_{b4(2B)}$ = 175 s^{-1}		[9–11]
Reac18	$Glu \xrightarrow{k_f} Glu_{\deg rad}$	k_f = 833 s^{-1}	Glutamate degradation	[81]
Reac19	$Ca^{2+} + UB \underset{k_b}{\overset{k_f}{\rightleftharpoons}} (Ca^{2+})UB$	k_f = 10 µmol^{-1}.L.s^{-1}<br>k_b = 100 s^{-1}	Interaction of Ca^{2+} with UB.	This paper
Reac20	$Ca^{2+} + CaM_{I,II} \underset{k_b}{\overset{k_f}{\rightleftharpoons}} (Ca^{2+})_I.CaM_{II}$	k_f = 750 µmol^{-1}.L.s^{-1}<br>k_b = 50000 s^{-1}	Reversible formation of the complex CaM with Ca^{2+} associated to the Ca^{2+}-binding	[32]

			site I, located in the N-terminal of CaM, while the Ca^{2+}-binding site II is empty. For the reactions of interaction between CaM and Ca^{2+}, in the notation $(Ca^{2+})_xCaM_y$, the terms x and y indicate the Ca^{2+}-binding sites filled with	

			Ca^{2+} and empty, respectively. Sites that were not mentioned explicitly indicated that their state (filled with Ca^{2+} or empty) do not interfere with the reactions been described. See the documentation of BioNetGen	

			for further information on rule-based modelling [26].	
Reac21	$Ca^{2+} + CaM_{I,II} \underset{k_b}{\overset{k_f}{\rightleftharpoons}} (Ca^{2+})_{II}.CaM_{I}$	k_f = 750 µmol^{-1}.L.s^{-1}<br>k_b = 50000 s^{-1}	Reversible formation of the complex CaM with Ca^{2+} associated to the Ca^{2+}-binding site II, located in CaM N-terminal with the Ca^{2+}-binding site I empty.	[32]

Reac22	$Ca^{2+} + (Ca^{2+})_I.CaM_{II} \underset{k_b}{\overset{k_f}{\rightleftharpoons}} (Ca^{2+})_{I,II}.CaM$	k_f = 750 µmol^{-1}.L.s^{-1}<br>k_b = 625 s^{-1}	Reversible formation of the complex CaM with Ca^{2+} associated to the Ca^{2+}-binding site II, located in the N-terminal of CaM, with the Ca^{2+}-binding site I filled.	[32]
Reac23	$Ca^{2+} + (Ca^{2+})_{II}.CaM_{I} \underset{k_b}{\overset{k_f}{\rightleftharpoons}} (Ca^{2+})_{I,II}.CaM$	k_f = 750 µmol^{-1}.L.s^{-1}<br>k_b = 625 s^{-1}	Reversible formation of the complex CaM with Ca^{2+}	[32]

			associated to the Ca^{2+}-binding site I, located in the N-terminal, with the Ca^{2+}-binding site II filled.	
Reac24	$Ca^{2+} + CaM_{III,IV} \underset{k_b}{\overset{k_f}{\rightleftharpoons}} (Ca^{2+})_{III}.CaM_{IV}$	k_f = 800 µmol^{-1}.L.s^{-1}<br>k_b = 20000 s^{-1}	Reversible formation of the complex CaM with Ca^{2+} associated to the Ca^{2+}-binding site III, located in the C-terminal	

			of CaM, with the Ca^{2+}-binding site IV empty.	
Reac25	$Ca^{2+} + CaM_{III,IV} \underset{k_b}{\overset{k_f}{\rightleftharpoons}} (Ca^{2+})_{IV}.CaM_{III}$	k_f = 204 µmol^{-1}.L.s^{-1}<br>k_b = 5115 s^{-1}	Reversible formation of the complex CaM with Ca^{2+} associated to the Ca^{2+}-binding site IV with the Ca^{2+}-binding site III empty.	[32]
Reac26	$Ca^{2+} + (Ca^{2+})_{III}.CaM_{IV} \underset{k_b}{\overset{k_f}{\rightleftharpoons}} (Ca^{2+})_{III,IV}.CaM$	k_f = 204 µmol^{-1}.L.s^{-1}<br>k_b = 25.575 s^{-1}	Reversible formation of the complex CaM	[32]

			with Ca^{2+} associated to the Ca^{2+}-binding site IV with the Ca^{2+}-binding site III filled.	
Reac27	$Ca^{2+} + (Ca^{2+})_{IV}.CaM_{III} \underset{k_b}{\overset{k_f}{\rightleftharpoons}} (Ca^{2+})_{III,IV}.CaM$	k_f = 800 µmol^{-1}.L.s^{-1}<br>k_b = 100 s^{-1}	Reversible formation of the complex CaM with Ca^{2+} associated to the Ca^{2+}-binding site III with the Ca^{2+}-binding site IV filled.	32

Reac28	$Ca^{2+} + PMCA \underset{k_b}{\overset{k_f}{\rightleftharpoons}} (Ca^{2+})PMCA$<br>$(Ca^{2+})PMCA \xrightarrow{k_{cat}} PMCA$	k_f = 55 µmol^{-1}.L.s^{-1}<br>k_b = 250 s^{-1}<br>k_{cat} = 30 µmol.L^{-1}.s^{-1}	Basal rate of Ca^{2+} efflux through PMCA.	[33,34]
Reac29	$PMCA + (Ca^{2+})_C.CaM \underset{k_b}{\overset{k_f}{\rightleftharpoons}} PMCA.(Ca^{2+})_C.CaM$	k_f = 0.2 µmol^{-1}.L.s^{-1}<br>k_b = 0.002 s^{-1}	Interaction between PMCA and CaM associated to Ca^{2+} (one or two ions) only in the Ca^{2+}-binding sites located at its C-terminal, indicated by the term $(Ca^{2+})_C$.CaM.	[34,38,39]

Reac30	$PMCA + (Ca^{2+})_{N,C}.CaM \underset{k_b}{\overset{k_f}{\rightleftharpoons}} PMCA.(Ca^{2+})_{N,C}.CaM$	$k_f = 0.2$ µmol^{-1}.L.s^{-1}<br>$k_b = 0.0012$ s^{-1}	Interaction between PMCA and CaM associated to three or four Ca^{2+}, indicated by the term $(Ca^{2+})_{N,C}$.CaM.	[34,38,39]
Reac31	$Ca^{2+} + (Ca^{2+})_C.CaM.PMCA \underset{k_b}{\overset{k_f}{\rightleftharpoons}} (Ca^{2+}).(Ca^{2+})_C.CaM.PMCA$<br>$(Ca^{2+}).(Ca^{2+})_C.CaM.PMCA \xrightarrow{k_{cat}} (Ca^{2+})_C.CaM.PMCA$	$k_f = 55$ µmol^{-1}.L.s^{-1}<br>$k_b = 250$ s^{-1}<br>$k_{cat} = 30$ µmol.L^{-1}.s^{-1}	Ca^{2+} efflux	[33,34]
Reac32	$Ca^{2+} + (Ca^{2+})_{N,C}.CaM.PMCA \underset{k_b}{\overset{k_f}{\rightleftharpoons}} (Ca^{2+}).(Ca^{2+})_{N,C}.CaM.PMCA$<br>$(Ca^{2+}).(Ca^{2+})_{N,C}.CaM.PMCA \xrightarrow{k_{cat}} (Ca^{2+})_{N,C}.CaM.PMCA$	$k_f = 50$ µmol^{-1}.L.s^{-1}<br>$k_b = 7.5$ s^{-1}<br>$k_{cat} = 30$ µmol.L^{-1}.s^{-1}	Ca^{2+} efflux	[33,34]
Reac33	$Ca^{2+} + (Ca^{2+})_C.CaM_{I,II}.PMCA \underset{k_b}{\overset{k_f}{\rightleftharpoons}} (Ca^{2+})_{I,C}.CaM_{II}.PMCA$	$k_f = 750$ µmol^{-1}.L.s^{-1}	Binding of Ca^{2+}	[32,34,49]

		$k_b = 12500\ s^{-1}$	to CaM associated to PMCA. The term $(Ca^{2+})_C.CaM_{I,II}$ indicates the presence of Ca^{2+} (one or two ions) bound to the Ca^{2+}-binding sites of the C-terminal, with no Ca^{2+} associated to the Ca^{2+}-binding sites of	

			the N-terminal.	
Reac34	$Ca^{2+} + (Ca^{2+})_C.CaM_{I,II}.PMCA \underset{k_b}{\overset{k_f}{\rightleftharpoons}} (Ca^{2+})_{II,C}.CaM_I.PMCA$	k_f = 750 µmol^{-1}.L.s^{-1}<br>k_b = 12500 s^{-1}	Binding of Ca^{2+} to CaM associated to PMCA.	[32,34,49]
Reac35	$Ca^{2+} + (Ca^{2+})_{I,C}.CaM_{II}.PMCA \underset{k_b}{\overset{k_f}{\rightleftharpoons}} (Ca^{2+})_{I,II,C}.CaM.PMCA$	k_f = 750 µmol^{-1}.L.s^{-1}<br>k_b = 162.5 s^{-1}	Cooperative binding of Ca^{2+} to CaM associated to PMCA.	[32,34,49]
Reac36	$Ca^{2+} + (Ca^{2+})_{II,C}.CaM_I.PMCA \underset{k_b}{\overset{k_f}{\rightleftharpoons}} (Ca^{2+})_{I,II,C}.CaM.PMCA$	k_f = 750 µmol^{-1}.L.s^{-1}<br>k_b = 162.5 s^{-1}	Cooperative binding of Ca^{2+} to CaM associated to PMCA.	[32,34,49]

Reac37	$Ca^{2+} + (Ca^{2+})_{III}.CaM_{IV}.PMCA \underset{k_b}{\overset{k_f}{\rightleftharpoons}} (Ca^{2+})_{III,IV}.CaM.PMCA$	k_f = 204 µmol^{-1}.L.s^{-1}<br>k_b = 6.39375 s^{-1}	Cooperative binding of Ca^{2+} to the Ca^{2+}-binding site IV of CaM associated to PMCA.	[32,34,49]
Reac38	$Ca^{2+} + (Ca^{2+})_{IV}.CaM_{III}.PMCA \underset{k_b}{\overset{k_f}{\rightleftharpoons}} (Ca^{2+})_{III,IV}.CaM.PMCA$	k_f = 800 µmol^{-1}.L.s^{-1}<br>k_b = 25 s^{-1}	Cooperative binding of Ca^{2+} to the Ca^{2+}-binding site III of CaM associated to PMCA.	[32,34,49]

Reac39	$Ca^{2+} + NCX \underset{k_b}{\overset{k_f}{\rightleftharpoons}} (Ca^{2+})NCX$<br>$(Ca^{2+})NCX \xrightarrow{k_{cat}} NCX$	k_f = 250 µmol^{-1}.L.s^{-1}<br>k_b = 100 s^{-1}<br>k_{cat} = 2400 s^{-1}	Non-conservative efflux of Ca^{2+} from the cytosol catalyzed by NCX.	[32]
Reac40	$leak \xrightarrow{k_{leak}} leak + Ca^{2+}$	k_{leak} = 400 µmol^{-1}.L.s^{-1}	First order reaction of a constant leak of Ca^{2+} to the cytosol to sustain the basal [Ca^{2+}].	This paper
Reac41	$Ca^{2+} + CNB_{I,II} \underset{k_b}{\overset{k_f}{\rightleftharpoons}} (Ca^{2+})_I.CNB_{II}$	k_f = 6.4 µmol^{-1}.L.s^{-1}<br>k_b = 0.03 s^{-1}	Reversible interaction	[5,41]

			between Ca^{2+} and the subunit CNB of CaN. The term I, II, III and IV denote the Ca^{2+}-binding sites of CNB. In the notation $(Ca^{2+})_xCNB_y$, the terms x and y indicate the Ca^{2+}-binding sites filled with Ca^{2+} and empty, respectively.	

			The interaction of Ca^{2+} to the Ca^{2+}- binding sites located at each terminal of CNB was considered independent of the state of the Ca^{2+}-binding sites of the other terminal.	
Reac42	$Ca^{2+} + (Ca^{2+})_I.CNB_{II} \underset{k_b}{\overset{k_f}{\rightleftharpoons}} (Ca^{2+})_{I,II}.CNB$	k_f = 6.4 µmol^{-1}.L.s^{-1}<br>k_b = 0.0018 s^{-1}		[5,41]
Reac43	$Ca^{2+} + CNB_{III,IV} \underset{k_b}{\overset{k_f}{\rightleftharpoons}} (Ca^{2+})_{III}.CNB_{IV}$	k_f = 0.09 µmol^{-1}.L.s^{-1}		[5,41]

		$k_b = 0.05\ s^{-1}$		
Reac44	$Ca^{2+} + (Ca^{2+})_{III}.CNB_{IV} \underset{k_b}{\overset{k_f}{\rightleftharpoons}} (Ca^{2+})_{III,IV}.CNB$	$k_f = 0.09\ \mu mol^{-1}.L.s^{-1}$<br>$k_b = 0.025\ s^{-1}$		5,41
Reac45	$CNA + (Ca^{2+})_{N,C}.CaM \underset{k_b}{\overset{k_f}{\rightleftharpoons}} (Ca^{2+})_{N,C}.CaM.CNA$	$k_f = 46\ \mu mol^{-1}.L.s^{-1}$<br>$k_b = 0.0012\ s^{-1}$	Interaction between the subunit CNA of CaN with CaM associated to at least one Ca^{2+} bound to each one of its globular domain. The notation $(Ca^{2+})_{N,C}.CaM$ indicated that	45

			both the C and the N-terminal domain of CaM have Ca^{2+} associated to it.	
Reac46	$CNA + (Ca^{2+})_C.CaM \underset{k_b}{\overset{k_f}{\rightleftharpoons}} (Ca^{2+})_C.CaM.CNA$	k_f = 46 µmol^{-1}.L.s^{-1}<br>k_b = 46 s^{-1}	Interaction between the subunit CNA of CaN with CaM associated to Ca^{2+} (one or two ions) to the Ca^{2+}-binding sites located in its C-terminal.	[45,46]

Reac47	$CNA + (Ca^{2+})_N.CaM \underset{k_b}{\overset{k_f}{\rightleftharpoons}} (Ca^{2+})_N.CaM.CNA$	k_f = 46 µmol^{-1}.L.s^{-1}<br>k_b = 322 s^{-1}	Interaction between the subunit CNA of CaN with CaM associated to Ca^{2+} (one or two ions) in the Ca^{2+}-binding sites located in its N-terminal.	[45,46]
Reac48	$Ca^{2+} + CaM_{I,II}.CNA \underset{k_b}{\overset{k_f}{\rightleftharpoons}} (Ca^{2+})_I.CaM_{II}.CNA$	k_f = 750 µmol^{-1}.L.s^{-1}<br>k_b = 950 s^{-1}	Reversible interaction of the complex CaM associated to the subunit CNA of	[32,46]

			CaN with Ca^{2+}.	
Reac49	$Ca^{2+} + CaM_{I,II}.CNA \underset{k_b}{\overset{k_f}{\rightleftharpoons}} (Ca^{2+})_{II}.CaM_I.CNA$	$k_f = 750\ \mu mol^{-1}.L.s^{-1}$<br>$k_b = 950\ s^{-1}$	Reversible interaction of the complex CaM associated to the subunit CNA of CaN with Ca^{2+}.	[32,46]
Reac50	$Ca^{2+} + (Ca^{2+})_I.CaM_{II}.CNA \underset{k_b}{\overset{k_f}{\rightleftharpoons}} (Ca^{2+})_{I,II}.CaM.CNA$	$k_f = 750\ \mu mol^{-1}.L.s^{-1}$<br>$k_b = 11.9\ s^{-1}$	Reversible interaction of the complex CaM associated to the subunit CNA of CaN with Ca^{2+}.	[32,46]
Reac51	$Ca^{2+} + (Ca^{2+})_{II}.CaM_I.CNA \underset{k_b}{\overset{k_f}{\rightleftharpoons}} (Ca^{2+})_{I,II}.CaM.CNA$	$k_f = 750\ \mu mol^{-1}.L.s^{-1}$<br>$k_b = 11.9\ s^{-1}$	Reversible interaction of the	[32,46]

			complex CaM associated to the subunit CNA of CaN with Ca^{2+}.	
Reac52	$Ca^{2+} + CaM_{III,IV}.CNA \underset{k_b}{\overset{k_f}{\rightleftharpoons}} (Ca^{2+})_{III}.CaM_{IV}.CNA$	k_f = 800 $\mu mol^{-1}.L.s^{-1}$<br>k_b = 160 s^{-1}	Reversible interaction of the complex CaM associated to the subunit CNA of CaN with Ca^{2+}.	[32,46]
Reac53	$Ca^{2+} + CaM_{III,IV}.CNA \underset{k_b}{\overset{k_f}{\rightleftharpoons}} (Ca^{2+})_{IV}.CaM_{III}.CNA$	k_f = 204 $\mu mol^{-1}.L.s^{-1}$<br>k_b = 48 s^{-1}	Reversible interaction of the complex CaM associated to the subunit CNA of	[32,46]

			CaN with Ca^{2+}.	
Reac54	$Ca^{2+} + (Ca^{2+})_{III}.CaM_{IV}.CNA \underset{k_b}{\overset{k_f}{\rightleftharpoons}} (Ca^{2+})_{III,IV}.CaM.CNA$	$k_f = 204\ \mu mol^{-1}.L.s^{-1}$ $k_b = 0.24\ s^{-1}$	Reversible interaction of the complex CaM associated to the subunit CNA of CaN with Ca^{2+}	[32,46]
Reac55	$Ca^{2+} + (Ca^{2+})_{IV}.CaM_{III}.CNA \underset{k_b}{\overset{k_f}{\rightleftharpoons}} (Ca^{2+})_{III,IV}.CaM.CNA$	$k_f = 800\ \mu mol^{-1}.L.s^{-1}$ $k_b = 0.8\ s^{-1}$	Reversible interaction of the complex CaM associated to the subunit CNA of CaN with Ca^{2+}	[32,46]
Reac56	$(Ca^{2+})_{I,II,III,IV}.CaM + AC \underset{k_b}{\overset{k_f}{\rightleftharpoons}} (Ca^{2+})_{I,II,III,IV}.CaM.AC$	$k_f = 0.2\ \mu mol^{-1}.L.s^{-1}$ $k_b = 0.01\ s^{-1}$	Binding and unbinding of AC	[51,53]

			to CaM fully loaded with Ca^{2+}.	
Reac57	$(Ca^{2+})_{II,III,IV}.CaM_{I} + AC \underset{k_b}{\overset{k_f}{\rightleftharpoons}} (Ca^{2+})_{II,III,IV}.CaM_{I}.AC$	k_f = 0.2 µmol^{-1}.L.s^{-1}<br>k_b = 0.02 s^{-1}	Binding and unbinding of AC to CaM with Ca^{2+} bound to its Ca^{2+}-binding sites II, III and IV.	[51,53]
Reac58	$(Ca^{2+})_{I,II,IV}.CaM_{III} + AC \underset{k_b}{\overset{k_f}{\rightleftharpoons}} (Ca^{2+})_{I,II,IV}.CaM_{III}.AC$	k_f = 0.2 µmol^{-1}.L.s^{-1}<br>k_b = 0.05 s^{-1}	Binding and unbinding of AC to CaM with Ca^{2+} bound to its Ca^{2+}-binding	[51,53]

			sites I, II and IV.	
Reac59	$(Ca^{2+})_{I,III,IV}.CaM_{II} + AC \underset{k_b}{\overset{k_f}{\rightleftharpoons}} (Ca^{2+})_{I,III,IV}.CaM_{II}.AC$	k_f = 0.2 µmol^{-1}.L.s^{-1}<br>k_b = 0.15 s^{-1}	Binding and unbinding of AC to CaM with Ca^{2+} bound to its Ca^{2+}-binding sites I, III and IV.	[51,53]
Reac60	$(Ca^{2+})_{I,II,III}.CaM_{IV} + AC \underset{k_b}{\overset{k_f}{\rightleftharpoons}} (Ca^{2+})_{I,II,III}.CaM_{IV}.AC$	k_f = 0.2 µmol^{-1}.L.s^{-1}<br>k_b = 0.3 s^{-1}	Binding and unbinding of AC to CaM with Ca^{2+} bound to its Ca^{2+}-binding sites I, II and III.	[51,53]
Reac61	$(Ca^{2+})_{I,IV}.CaM_{II,III} + AC \underset{k_b}{\overset{k_f}{\rightleftharpoons}} (Ca^{2+})_{I,IV}.CaM_{II,III}.AC$	k_f = 0.2 µmol^{-1}.L.s^{-1}	Binding and	[51,53]

		$k_b = 0.75\ s^{-1}$	unbinding of AC to CaM partially loaded with Ca^{2+} bound to its Ca^{2+}-binding sites I and IV	
Reac62	$(Ca^{2+})_{I,II}.CaM_{III,IV} + AC \underset{k_b}{\overset{k_f}{\rightleftharpoons}} (Ca^{2+})_{I,II}.CaM_{III,IV}.AC$	$k_f = 0.2\ \mu mol^{-1}.L.s^{-1}$ $k_b = 1.5\ s^{-1}$	Binding and unbinding of AC to CaM partially loaded with Ca^{2+} bound to its Ca^{2+}-binding sites I and II.	[51,53]
Reac63	$(Ca^{2+})_{I,III}.CaM_{II,IV} + AC \underset{k_b}{\overset{k_f}{\rightleftharpoons}} (Ca^{2+})_{I,III}.CaM_{II,IV}.AC$	$k_f = 0.2\ \mu mol^{-1}.L.s^{-1}$ $k_b = 4.5\ s^{-1}$	Binding and unbinding of AC	[51,53]

			to CaM partially loaded with Ca^{2+} bound to its Ca^{2+}-binding sites I and III.	
Reac64	$(Ca^{2+})_{II,IV}.CaM_{I,III} + AC \underset{k_b}{\overset{k_f}{\rightleftharpoons}} (Ca^{2+})_{II,IV}.CaM_{I,III}.AC$	$k_f = 0.2\ \mu mol^{-1}.L.s^{-1}$<br>$k_b = 0.1\ s^{-1}$	Binding and unbinding of AC to CaM partially loaded with Ca^{2+} bound to its Ca^{2+}-binding sites II and IV.	[51,53]
Reac65	$(Ca^{2+})_{III,IV}.CaM_{I,II} + AC \underset{k_b}{\overset{k_f}{\rightleftharpoons}} (Ca^{2+})_{III,IV}.CaM_{I,II}.AC$	$k_f = 0.2\ \mu mol^{-1}.L.s^{-1}$<br>$k_b = 0.3\ s^{-1}$	Binding and unbinding of AC to CaM partially	[51,53]

			loaded with Ca^{2+} bound to its Ca^{2+}-binding sites III and IV.	
Reac66	$(Ca^{2+})_{II,III}.CaM_{I,IV} + AC \underset{k_b}{\overset{k_f}{\rightleftharpoons}} (Ca^{2+})_{II,III}.CaM_{I,IV}.AC$	k_f = 0.2 µmol^{-1}.L.s^{-1}<br>k_b = 0.6 s^{-1}	Binding and unbinding of AC to CaM partially loaded with Ca^{2+} bound to its Ca^{2+}-binding sites II and III.	[51,53]
Reac67	$(Ca^{2+})_{III}.CaM_{I,II,IV} + AC \underset{k_b}{\overset{k_f}{\rightleftharpoons}} (Ca^{2+})_{III}.CaM_{I,II,IV}.AC$	k_f = 0.2 µmol^{-1}.L.s^{-1}<br>k_b = 9 s^{-1}	Binding and unbinding of AC to CaM partially loaded with Ca^{2+}	[51,53]

			bound to its Ca^{2+}-binding site III.	
Reac68	$(Ca^{2+})_{II}.CaM_{I,III,IV} + AC \underset{k_b}{\overset{k_f}{\rightleftharpoons}} (Ca^{2+})_{II}.CaM_{I,III,IV}.AC$	$k_f = 0.2\ \mu mol^{-1}.L.s^{-1}$ $k_b = 3\ s^{-1}$	Binding and unbinding of AC to CaM partially loaded with Ca^{2+} bound to its Ca^{2+}-binding site II.	[51,53]
Reac69	$(Ca^{2+})_{IV}.CaM_{I,II,III} + AC \underset{k_b}{\overset{k_f}{\rightleftharpoons}} (Ca^{2+})_{IV}.CaM_{I,II,III}.AC$	$k_f = 0.2\ \mu mol^{-1}.L.s^{-1}$ $k_b = 1.5\ s^{-1}$	Binding and unbinding of AC to CaM partially loaded with Ca^{2+} bound to its	[51,53]

			Ca^{2+}-binding site IV	
Reac70	$(Ca^{2+})_I.CaM_{II,III,IV} + AC \underset{k_b}{\overset{k_f}{\rightleftharpoons}} (Ca^{2+})_I.CaM_{II,III,IV}.AC$	k_f = 0.2 µmol^{-1}.L.s^{-1}<br>k_b = 22.5 s^{-1}	Binding and unbinding of AC to CaM partially loaded with Ca^{2+} bound to its Ca^{2+}-binding site I.	[51,53]
Reac71	$Ca^{2+} + (Ca^{2+})_I.CaM_{II}.AC \underset{k_b}{\overset{k_f}{\rightleftharpoons}} (Ca^{2+})_{I,II}.CaM.AC$	k_f = 750 µmol^{-1}.L.s^{-1}<br>k_b = 1.25 s^{-1}	Cooperative association and dissociation of Ca^{2+} to CaM bound to AC. The presence or	[32,52]

			absence of Ca^{2+} associated to the Ca^{2+}-binding sites in the C-terminal of CaM does not interfere in these reactions.	
Reac72	$Ca^{2+} + (Ca^{2+})_{II}.CaM_{I}.AC \underset{k_b}{\overset{k_f}{\rightleftharpoons}} (Ca^{2+})_{I,II}.CaM.AC$	k_f = 750µmol^{-1}.L.s^{-1}<br>k_b = 156.25 s^{-1}	Association and dissociation of Ca^{2+} to CaM bound to AC.	[32,52]
Reac73	$Ca^{2+} + (Ca^{2+})_{N}.CaM_{III,IV}.AC \underset{k_b}{\overset{k_f}{\rightleftharpoons}} (Ca^{2+})_{N,III}.CaM_{IV}.AC$	k_f = 800 µmol^{-1}.L.s^{-1}<br>k_b = 80 s^{-1}	Association and dissociation of Ca^{2+} to CaM	[32,52]

			bound to AC. The term $(Ca^{2+})_N.CaM$ indicates that CaM is partially bound to Ca^{2+} in its N-terminal.	
Reac74	$Ca^{2+} + (Ca^{2+})_N.CaM.AC \underset{k_b}{\overset{k_f}{\rightleftharpoons}} (Ca^{2+})_{N,IV}.CaM.AC$	k_f = 204 µmol^{-1}.L.s^{-1}<br>k_b = 20.46 s^{-1}	Association and dissociation of Ca^{2+} to CaM bound to AC.	[32,52]
Reac75	$Ca^{2+} + (Ca^{2+})_C.CaM_{I,II}.AC \underset{k_b}{\overset{k_f}{\rightleftharpoons}} (Ca^{2+})_{I,C}.CaM_{II}.AC$	k_f = 750 µmol^{-1}.L.s^{-1}<br>k_b = 12500 s^{-1}	Association and dissociation of Ca^{2+} to CaM bound to AC.	[32,52]

Reac76	$Ca^{2+} + (Ca^{2+})_{C}.CaM_{I,II}.AC \underset{k_b}{\overset{k_f}{\rightleftharpoons}} (Ca^{2+})_{II,C}.CaM_{I}.AC$	$k_f = 750\ \mu mol^{-1}.L.s^{-1}$ $k_b = 100\ s^{-1}$	Association and dissociation of Ca^{2+} to CaM bound to AC.	[32,52]
Reac77	$Ca^{2+} + (Ca^{2+})_{III}.CaM_{IV}.AC \underset{k_b}{\overset{k_f}{\rightleftharpoons}} (Ca^{2+})_{III,IV}.CaM.AC$	$k_f = 204\ \mu mol^{-1}.L.s^{-1}$ $k_b = 0.102\ s^{-1}$	Cooperative association and dissociation of Ca^{2+} to CaM bound to AC.	[32,52]
Reac78	$Ca^{2+} + (Ca^{2+})_{IV}.CaM_{III}.AC \underset{k_b}{\overset{k_f}{\rightleftharpoons}} (Ca^{2+})_{III,IV}.CaM.AC$	$k_f = 800\ \mu mol^{-1}.L.s^{-1}$ $k_b = 0.4\ s^{-1}$	Cooperative association and dissociation of Ca^{2+} to CaM bound to AC.	[32,52]
Reac79	$Ca^{2+} + AC \underset{k_b}{\overset{k_f}{\rightleftharpoons}} (Ca^{2+}).AC$	$k_f = 0.1\ \mu mol^{-1}.L.s^{-1}$	Inhibitory	This paper,

		$k_b = 8\ s^{-1}$	interaction of Ca^{2+} to AC. These reactions occur with the same rate constants in the presence or absence of CaM associated to AC.	estimated from [55]
Reac80	$(Ca^{2+})_N.CaM_{III,IV} + PDE1 \underset{k_b}{\overset{k_f}{\rightleftharpoons}} (Ca^{2+})_N.CaM_{III,IV}.PDE1$	$k_f = 1\ \mu mol^{-1}.L.s^{-1}$ $k_b = 0.05\ s^{-1}$	Interaction of PDE1 with CaM partially loaded with Ca^{2+} bound to the Ca^{2+}	[57,59,61]

			binding sites of its N-terminal.	
Reac81	$(Ca^{2+})_C.CaM_{I,II} + PDE1 \underset{k_b}{\overset{k_f}{\rightleftharpoons}} (Ca^{2+})_C.CaM_{I,II}.PDE1$	k_f = 1 µmol^{-1}.L.s^{-1}<br>k_b = 0.1 s^{-1}	Interaction of PDE1 with CaM partially loaded with Ca^{2+} bound to the Ca^{2+} binding sites of its C-terminal.	[57,59,61]
Reac82	$(Ca^{2+})_{N,C}.CaM + PDE1 \underset{k_b}{\overset{k_f}{\rightleftharpoons}} (Ca^{2+})_{N,C}.CaM.PDE1$	k_f = 1 µmol^{-1}.L.s^{-1}<br>k_b = 0.05 s^{-1}	Interaction of PDE1 with CaM partially loaded with Ca^{2+} bound to one of the Ca^{2+} binding	[57,59,61]

			sites of its C-terminal and one of its N-terminal.	
Reac83	$(Ca^{2+})_{N,III,IV}.CaM + PDE1 \underset{k_b}{\overset{k_f}{\rightleftharpoons}} (Ca^{2+})_{N,III,IV}.CaM.PDE1$	k_f = 1 µmol^{-1}.L.s^{-1}<br>k_b = 0.01 s^{-1}	Interaction of PDE1 with CaM partially loaded with Ca^{2+} bound to the two Ca^{2+} binding sites of its C-terminal and only one of the sites of its N-terminal.	[57,59,61]
Reac84	$(Ca^{2+})_{I,II,C}.CaM + PDE1 \underset{k_b}{\overset{k_f}{\rightleftharpoons}} (Ca^{2+})_{I,II,C}.CaM.PDE1$	k_f = 1 µmol^{-1}.L.s^{-1}	Interaction of	[57,59,61]

		$k_b = 0.0001\ s^{-1}$	PDE1 with CaM partially loaded with Ca^{2+} bound to the Ca^{2+} binding sites of its C-terminal (one or two ions) and two of the sites of its N-terminal. The same rates were used for the interaction of PDE1 with CaM fully saturated	

			with Ca^{2+}.	
Reac85	$Ca^{2+} + (Ca^{2+})_C.CaM_{I,II}.PDE1 \underset{k_b}{\overset{k_f}{\rightleftharpoons}} (Ca^{2+})_{I,C}.CaM_{II}.PDE1$	k_f = 750 µmol^{-1}.L.s^{-1}<br>k_b = 2000 s^{-1}	Binding of Ca^{2+} to the Ca^{2+}-binding site I of CaM associated to PDE1.	[32,62]
Reac86	$Ca^{2+} + (Ca^{2+})_C.CaM_{I,II}.PDE1 \underset{k_b}{\overset{k_f}{\rightleftharpoons}} (Ca^{2+})_{II,C}.CaM_{I}.PDE1$	k_f = 750 µmol^{-1}.L.s^{-1}<br>k_b = 2000 s^{-1}	Binding of Ca^{2+} to the Ca^{2+}-binding site II of CaM associated to PDE1.	[32,62]
Reac87	$Ca^{2+} + (Ca^{2+})_N.CaM_{III,IV}.PDE1 \underset{k_b}{\overset{k_f}{\rightleftharpoons}} (Ca^{2+})_{III,N}.CaM_{IV}.PDE1$	k_f = 800 µmol^{-1}.L.s^{-1}<br>k_b = 800 s^{-1}	Binding of Ca^{2+} to the Ca^{2+}-binding site III of CaM	[32,62]

			associated to PDE1.	
Reac88	$Ca^{2+} + (Ca^{2+})_N.CaM_{III,IV}.PDE1 \underset{k_b}{\overset{k_f}{\rightleftharpoons}} (Ca^{2+})_{IV,N}.CaM_{III}.PDE1$	k_f = 204 µmol^{-1}.L.s^{-1} k_b = 204 s^{-1}	Binding of Ca^{2+} to the Ca^{2+}-binding site IV of CaM associated to PDE1.	[32,62]
Reac89	$Ca^{2+} + (Ca^{2+})_I.CaM_{II}.PDE1 \underset{k_b}{\overset{k_f}{\rightleftharpoons}} (Ca^{2+})_{I,II}.CaM.PDE1$	k_f = 750 µmol^{-1}.L.s^{-1} k_b = 25 s^{-1}	Cooperative binding of Ca^{2+} to the Ca^{2+}-binding site II of CaM associated to PDE1.	[32,62]
Reac90	$Ca^{2+} + (Ca^{2+})_{II}.CaM_I.PDE1 \underset{k_b}{\overset{k_f}{\rightleftharpoons}} (Ca^{2+})_{I,II}.CaM.PDE1$	k_f = 750 µmol^{-1}.L.s^{-1}	Cooperative	[32,62]

		$k_b = 25\ s^{-1}$	binding of Ca^{2+} to the Ca^{2+}-binding site I of CaM associated to PDE1.	
Reac91	$Ca^{2+} + (Ca^{2+})_{IV}.CaM_{III}.PDE1 \underset{k_b}{\overset{k_f}{\rightleftharpoons}} (Ca^{2+})_{III,IV}.CaM.PDE1$	$k_f = 800$ µmol^{-1}.L.s^{-1}<br>$k_b = 4\ s^{-1}$	Cooperative binding of Ca^{2+} to the Ca^{2+}-binding site III of CaM associated to PDE1.	[32,62]
Reac92	$Ca^{2+} + (Ca^{2+})_{III}.CaM_{IV}.PDE1 \underset{k_b}{\overset{k_f}{\rightleftharpoons}} (Ca^{2+})_{III,IV}.CaM.PDE1$	$k_f = 204$ µmol^{-1}.L.s^{-1}<br>$k_b = 1.02\ s^{-1}$	Cooperative binding of Ca^{2+} to the Ca^{2+}-	[32,62]

			binding site IV of CaM associated to PDE1.	
Reac93	$AC \underset{k_b}{\overset{[ATP]\times k_f}{\rightleftharpoons}} AC.ATP$<br>$AC.ATP \xrightarrow{k_{cat}} AC + cAMP$	$k_f = 0.125\ \mu mol^{-1}.L.s^{-1}$<br>$k_b = 40\ s^{-1}$<br>$k_{cat} = 10\ s^{-1}$	[ATP] = 10 $mmol.L^{-1}$<br>Basal catalytic rates of AC estimated from experimental data obtained in absence of Ca^{2+}.	[55]
Reac94	$AC \underset{k_b}{\overset{[ATP]\times k_f}{\rightleftharpoons}} AC.ATP$<br>$AC.ATP \xrightarrow{k_{cat}} AC + cAMP$	$k_f = 1.25\ \mu mol^{-1}.L.s^{-1}$<br>$k_b = 400\ s^{-1}$<br>$k_{cat} = 50\ s^{-1}$	Conversion of ATP to cAMP catalyzed by AC	[51,55]

			bound to CaM with less than four Ca^{2+} associated to its structure. The rate constants were based on experimental reports that indicates that Ca^{2+} stimulates AC catalytic activity.	
Reac95	$(Ca^{2+})_{N,C}.CaM.AC \underset{k_b}{\overset{ATP \times k_f}{\rightleftharpoons}} (Ca^{2+})_{N,C}.CaM.AC.ATP$<br>$(Ca^{2+})_{N,C}.CaM.AC.ATP \xrightarrow{k_{cat}} (Ca^{2+})_{N,C}.CaM.AC + cAMP$	$k_f = 1.25\ \mu mol^{-1}.L.s^{-1}$<br>$k_b = 400\ s^{-1}$	[ATP] = 10 $mmol.L^{-1}$	[51,55]

		k_{cat} = 1000 s^{-1}	Conversion of ATP to cAMP catalyzed by AC bound to CaM fully saturated with Ca^{2+}. The rate constants were based on experimental reports that indicates that Ca^{2+} stimulates AC catalytic activity.	

Reac96	$(Ca^{2+})_{N,C}.CaM.PDE1 + cAMP \underset{k_b}{\overset{k_f}{\rightleftharpoons}} (Ca^{2+})_{N,C}.CaM.PDE1.cAMP$<br>$(Ca^{2+})_{N,C}.CaM.PDE1.cAMP \xrightarrow{k_{cat}} (Ca^{2+})_{N,C}.CaM.PDE1$	$k_f = 33\ \mu mol^{-1}.L.s^{-1}$<br>$k_b = 800\ s^{-1}$<br>$k_{cat} = 200\ s^{-1}$	Rate constants for the degradation of cAMP catalyzed by PDE1.	Estimated from[57,68]
Reac97	$cAMP \xrightarrow{k_{cat}}$	$k_{cat} = 200\ s^{-1}$	Constant rate of cAMP degradation implemented to counteract AC basal activity. The parameter were set to sustain the basal [cAMP] around	This paper

			100 $nmol.L^{-1}$	
Reac98	$C + RII\beta \underset{k_b}{\overset{k_f}{\rightleftharpoons}} (RII\beta)C$	$k_f = 2.1\ \mu mol^{-1}.L.s^{-1}$<br>$k_b = 3\ 10^{-4}\ s^{-1}$	Interaction between the subunits C and RIIβ in absence of cAMP	[8]
Reac99	$(RII\beta)C \xrightarrow{k_{cat}} (RII\beta^P)C$	$k_{cat} = 17.9\ s^{-1}$	Autophosphorylation of PKA. This reaction occurs with the same rate independently of the presence of cAMP bound to RIIβ.	[10]

Reac100	$C + RII\beta^{P} \underset{k_b}{\overset{k_f}{\rightleftharpoons}} (RII\beta^{P})C$	k_f = 0.038 µmol^{-1}.L.s^{-1}<br>k_b = 2.6 10^{-4} s^{-1}	Interaction between the subunits C and phosphorylated RIIβ (RIIβ^{P}) in absence of cAMP	[10]
Reac101	$cAMP + RII\beta_{CNB-A} \underset{k_b}{\overset{k_f}{\rightleftharpoons}} (cAMP)RII\beta_{CNB-A}$	k_f = 0.04 µmol^{-1}.L.s^{-1}<br>k_b = 0.013 s^{-1}	Interaction between cAMP and the CNB-A domain of a RIIβ subunit in the isolated (RIIβ)$_2$ dimer.	[74]
Reac102	$cAMP + RII\beta_{CNB-B} \underset{k_b}{\overset{k_f}{\rightleftharpoons}} (cAMP)RII\beta_{CNB-B}$	k_f = 0.015 µmol^{-1}.L.s^{-1}	Interaction	[74]

		$k_b = 0.0016\ s^{-1}$	between cAMP and the CNB-B domain of a RIIβ subunit in the isolated $(RII\beta)_2$ dimer.	
Reac103	$cAMP + \left(RII\beta_{CNB-A}\right)_2 C_2 \underset{k_b}{\overset{k_f}{\rightleftharpoons}} \left(cAMP\right).\left(RII\beta_{CNB-A}\right)_2 C_2$	$k_f = 0.35\ \mu mol^{-1}.L.s^{-1}$<br>$k_b = 0.33\ s^{-1}$	Interaction between cAMP and the CNB-B domain of the PKA holoenzyme. Based on experimental data, we	[74,75]

			assumed that the affinities of cAMP for the CNB domains of the tetrameric PKA are smaller than the affinities observed for the isolated R subunits[7,76].	
Reac104	$cAMP + \left(RII\beta_{CNB-B}\right)_2 C_2 \underset{k_b}{\overset{k_f}{\rightleftharpoons}} \left(cAMP\right).\left(RII\beta_{CNB-B}\right)_2 C_2$	k_f = 0.35 µmol^{-1}.L.s^{-1}<br>k_b = 0.105 s^{-1}	Interaction between cAMP and the CNB-B domain of the	[74,75]

			PKA holoenzyme. Based on experimental data, we assumed that the affinities of cAMP for the CNB domains of the tetrameric PKA are smaller than the affinities observed for the isolated R	

			subunits[7,76].	
Reac105	$\left(cAMP\right)_4\left(RII\beta_{CNB-A2,B2}\right)_2 C_2 \underset{k_b}{\overset{k_f}{\rightleftharpoons}} \left(cAMP\right)_4\left(RII\beta_{CNB-A2,B2}\right)_2 C + C$	k_f = 4.2 μmol^{-1}.L.s^{-1}<br>k_b = 0.009 s^{-1}<br>$k_{f(P)}$ = 0.076 μmol^{-1}.L.s^{-1}<br>$k_{(P)}$ = 0.0078 s^{-1}	Dissociation and association of a single C subunit from the tetrameric PKA full saturated with cAMP. The term CNB-A2,B2 refers to the CNB fully saturated with cAMP. The term (P) in the	[7,8]

			parameters indicates the rate constants for the phosphorylated PKA.	
Reac106	$(cAMP)_4\left(RII\beta_{CNB-A2,B2}\right)_2 C \underset{k_b}{\overset{k_f}{\rightleftharpoons}} (cAMP)_4\left(RII\beta_{CNB-A2,B2}\right)_2 + C$	k_f = 4.2 µmol^{-1}.L.s^{-1}<br>k_b = 0.9 s^{-1}<br>$k_{f(P)}$ = 0.076 µmol^{-1}.L.s^{-1}<br>$k_{(P)}$ = 0.78 s^{-1}	Cooperative dissociation and association of the second C subunit from the tetramer fully saturated with cAMP. The original parameters were	[7,8]

			altered to capture the cooperativity of PKA activation. See text for details.	
Reac107	$(cAMP)_3\left(RII\beta_{CNB-A2,B1}\right)_2 C_2 \underset{k_b}{\overset{k_f}{\rightleftharpoons}} (cAMP)_3\left(RII\beta_{CNB-A2,B1}\right)_2 C + C$	k_f = 4.2 µmol^{-1}.L.s^{-1}<br>k_b = 0.012 s^{-1}<br>$k_{f(P)}$ = 0.076 µmol^{-1}.L.s^{-1}<br>$k_{(P)}$ = 0.0104 s^{-1}	Dissociation of the first C subunit from the tetrameric PKA with two cAMP bound to the CNB-A domains and one cAMP associated to a	[7,8]

			CNB-B domain.	
Reac108	$(cAMP)_3(RII\beta_{CNB-A2,B1})_2 C \underset{k_b}{\overset{k_f}{\rightleftharpoons}} (cAMP)_3(RII\beta_{CNB-A2,B1})_2 + C$	k_f = 4.2 µmol^{-1}.L.s^{-1}<br>k_b = 0.012 s^{-1}<br>$k_{f(P)}$ = 0.076 µmol^{-1}.L.s^{-1}<br>$k_{(P)}$ = 0.0104 s^{-1}	Dissociation and association of the second C subunit from PKA with two cAMP bound to the CNB-A domains and one cAMP associated to a CNB-B domain.	[7,8]
Reac109	$(cAMP)_3(RII\beta_{CNB-A1,B2})_2 C_2 \underset{k_b}{\overset{k_f}{\rightleftharpoons}} (cAMP)_3(RII\beta_{CNB-A1,B2})_2 C + C$	k_f = 4.2 µmol^{-1}.L.s^{-1}<br>k_b = 1.2 10^{-5} s^{-1}<br>$k_{f(P)}$ = 0.076 µmol^{-1}.L.s^{-1}	Dissociation and association of the C subunit	[7,8]

		$k_{(P)} = 1.04\ 10^{-5}\ s^{-1}$	from PKA with two cAMP bound to the CNB-B domains and one cAMP associated to a CNB-A domain.	
Reac110	$(cAMP)_3 (RII\beta_{CNB-A1,B2})_2 C \underset{k_b}{\overset{k_f}{\rightleftharpoons}} (cAMP)_3 (RII\beta_{CNB-A1,B2})_2 + C$	$k_f = 4.2\ \mu mol^{-1}.L.s^{-1}$<br>$k_b = 1.2\ 10^{-5}\ s^{-1}$<br>$k_{f(P)} = 0.076\ \mu mol^{-1}.L.s^{-1}$<br>$k_{(P)} = 1.04\ 10^{-5}\ s^{-1}$	Dissociation and association of the second C subunit from PKA with two cAMP bound to the CNB-B domains and one	[7,8]

			cAMP associated to a CNB-A domain.	
Reac111	$\left(cAMP\right)_2\left(RII\beta_{CNB-A2,B}\right)_2C_2 \underset{k_b}{\overset{k_f}{\rightleftharpoons}} \left(cAMP\right)_2\left(RII\beta_{CNB-A2,B}\right)_2C+C$	k_f = 4.2 µmol^{-1}.L.s^{-1}<br>k_b = 0.012 s^{-1}<br>$k_{f(P)}$ = 0.076 µmol^{-1}.L.s^{-1}<br>$k_{(P)}$ = 0.0104 s^{-1}	Dissociation and association of the first C subunit from the tetrameric PKA with two cAMP bound to the CNB-A domains.	[7,8]
Reac112	$\left(cAMP\right)_2\left(RII\beta_{CNB-A2,B}\right)_2C \underset{k_b}{\overset{k_f}{\rightleftharpoons}} \left(cAMP\right)_2\left(RII\beta_{CNB-A2,B}\right)_2+C$	k_f = 4.2 µmol^{-1}.L.s^{-1}<br>k_b = 0.012 s^{-1}<br>$k_{f(P)}$ = 0.076 µmol^{-1}.L.s^{-1}	Dissociation and association of the second C	[7,8]

		$k_{(P)}$ = 0.0104 s^{-1}	subunit from PKA with two cAMP bound to the CNB-A domains.	
Reac113	$\left(cAMP\right)_2\left(RII\beta_{CNB-A,B2}\right)_2 C_2 \underset{k_b}{\overset{k_f}{\rightleftharpoons}} \left(cAMP\right)_2\left(RII\beta_{CNB-A,B2}\right)_2 C + C$	k_f = 4.2 µmol^{-1}.L.s^{-1}<br>k_b = 1.2 10^{-5} s^{-1}<br>$k_{f(P)}$ = 0.076 µmol^{-1}.L.s^{-1}<br>$k_{(P)}$ = 1.04 10^{-5} s^{-1}	Dissociation of a C subunit from the tetrameric PKA with two cAMP bound to the CNB-B domains.	[7,8]
Reac114	$\left(cAMP\right)_2\left(RII\beta_{CNB-A,B2}\right)_2 C \underset{k_b}{\overset{k_f}{\rightleftharpoons}} \left(cAMP\right)_2\left(RII\beta_{CNB-A,B2}\right)_2 + C$	k_f = 4.2 µmol^{-1}.L.s^{-1}<br>k_b = 1.2 10^{-5} s^{-1}<br>$k_{f(P)}$ = 0.076 µmol^{-1}.L.s^{-1}	Dissociation and association of the second C	[7,8]

		$k_{(P)} = 1.04\ 10^{-5}\ s^{-1}$	subunit from the tetrameric PKA with two cAMP bound to the CNB-B domains.	
Reac115	$\left(cAMP\right)_2\left(RII\beta_{CNB-A1,B1}\right)_2 C_2 \underset{k_b}{\overset{k_f}{\rightleftharpoons}} \left(cAMP\right)_2\left(RII\beta_{CNB-A1,B1}\right)_2 C + C$	$k_f = 2.1$ µmol^{-1}.L.s^{-1}<br>$k_b = 3\ 10^{-4}\ s^{-1}$<br>$k_{f(P)} = 0.038$ µmol^{-1}.L.s^{-1}<br>$k_{b(P)} = 2.6\ 10^{-4}\ s^{-1}$	Dissociation and association of a C subunit from the tetrameric PKA with one cAMP bound to one CNB-B domain and another to one	[7,8]

			CNB-A domain.	
Reac116	$\left(cAMP\right)_2\left(RII\beta_{CNB-A1,B1}\right)_2 C \underset{k_b}{\overset{k_f}{\rightleftharpoons}} \left(cAMP\right)_2\left(RII\beta_{CNB-A1,B1}\right)_2 + C$	k_f = 2.1 µmol^{-1}.L.s^{-1}<br>k_b = 3 10^{-4} s^{-1}<br>$k_{f(P)}$ = 0.038 µmol^{-1}.L.s^{-1}<br>$k_{b(P)}$ = 2.6 10^{-4} s^{-1}	Dissociation and association of the second C subunit from PKA with one cAMP bound to one CNB-B domain and another to one CNB-A domain.	[7,8]
Reac117	$\left(cAMP\right)_1\left(RII\beta_{CNB-A1,B}\right)_2 C_2 \underset{k_b}{\overset{k_f}{\rightleftharpoons}} \left(cAMP\right)_1\left(RII\beta_{CNB-A1,B}\right)_2 C + C$	k_f = 2.1 µmol^{-1}.L.s^{-1}<br>k_b = 3 10^{-4} s^{-1}<br>$k_{f(P)}$ = 0.038 µmol^{-1}.L.s^{-1}<br>$k_{b(P)}$ = 2.6 10^{-4} s^{-1}	Dissociation and association of a C subunit from the tetrameric	[7,8]

			PKA with one cAMP bound to the CNB-A domains.	
Reac118	$(cAMP)_1(RII\beta_{CNB-A1,B})_2C \underset{k_b}{\overset{k_f}{\rightleftharpoons}} (cAMP)_1(RII\beta_{CNB-A1,B})_2 + C$	k_f = 2.1 µmol^{-1}.L.s^{-1}<br>k_b = 3 10^{-4} s^{-1}<br>$k_{f(P)}$ = 0.038 µmol^{-1}.L.s^{-1}<br>$k_{b(P)}$ = 2.6 10^{-4} s^{-1}	Dissociation and association of the second C subunit from PKA with two cAMP bound to the CNB-A domains.	[7,8]
Reac119	$(cAMP)_1(RII\beta_{CNB-A,B1})_2C_2 \underset{k_b}{\overset{k_f}{\rightleftharpoons}} (cAMP)_1(RII\beta_{CNB-A,B1})_2C + C$	k_f = 2.1 µmol^{-1}.L.s^{-1}<br>k_b = 3 10^{-4} s^{-1}<br>$k_{f(P)}$ = 0.038 µmol^{-1}.L.s^{-1}	Dissociation and association of a C subunit from	[7,8]

		$k_{b(P)} = 2.6\ 10^{-4}\ s^{-1}$	the tetrameric PKA with one cAMP bound to the CNB-B domains.	
120	$(cAMP)_1 \left(RII\beta_{CNB-A,B1}\right)_2 C \underset{k_b}{\overset{k_f}{\rightleftharpoons}} (cAMP)_1 \left(RII\beta_{CNB-A,B1}\right)_2 + C$	$k_f = 2.1$ µmol^{-1}.L.s^{-1}<br>$k_b = 3\ 10^{-4}\ s^{-1}$<br>$k_{f(P)} = 0.038$ µmol^{-1}.L.s^{-1}<br>$k_{b(P)} = 2.6\ 10^{-4}\ s^{-1}$	Dissociation and association of the second C subunit from PKA with one cAMP bound to the CNB-B domains.	[7,8]
121	$C + Kemptide \underset{k_b}{\overset{k_f}{\rightleftharpoons}} C.Kemptide$<br>$C.Kemptide \xrightarrow{k_{cat}} C + Kemptide_P$	$k_f = 1.29$ µmol^{-1}.L.s^{-1}<br>$k_b = 350\ s^{-1}$	Rate constants for the	[82–84]

		k_b = 33.1 s^{-1}	phosphorylation of Kemptide catalyzed by the free subunit C of PKA. The same rate constants were used in the phosphorylation and dephosphorylation of subs by PKA and CaN	
